# Simple, Fast and Cheap Pb^2+^ and Hg^2+^ Detection in Liquid Media Using EGOFET-Based Aptasensors

**DOI:** 10.3390/ijms27219711

**Published:** 2026-10-29

**Authors:** Anna K. Keshek, Dmitriy A. Skvortsov, Elena Yu. Poimanova, Victor N. Orlov, Ilya P. Oleynikov, Margarita S. Stiazhkina, Askar D. Turashev, Elena G. Zavyalova, Elena V. Agina

**Affiliations:** 1 Enikolopov Institute of Synthetic Polymeric Materials of Russian Academy of Sciences, Profsoyuznaya Str. 70, 117393 Moscow, Russia; akeshek@list.ru (A.K.K.); dmitriiskv141@gmail.com (D.A.S.); poymanova@ispm.ru (E.Y.P.); zlenka2006@gmail.com (E.G.Z.); 2 Chemistry Department, Lomonosov Moscow State University, Leninskiye Gory 1/3, 119991 Moscow, Russia; 3 Department of Fundamental Physical and Chemical Engineering, Lomonosov Moscow State University, Leninskiye Gory 1/51, 119991 Moscow, Russia; 4 Belozersky Institute of Physico-Chemical Biology, Lomonosov Moscow State University, Leninskiye Gory 1/40, 119991 Moscow, Russia; orlovvn@belozersky.msu.ru (V.N.O.); oleynikov.biophys@gmail.com (I.P.O.); 5 Central Research Institute of Epidemiology, Federal Service for Surveillance on Consumer Rights Protection and Human Wellbeing Russian Federation, Novogireevskaya Str. 3a, 111123 Moscow, Russia; stiazhkina@cmd.su (M.S.S.); turashev@cmd.su (A.D.T.)

**Keywords:** aptamer, biosensor, EGOFET, lead, mercury, metal ions

## Abstract

Lead and mercury are among the most dangerous environmental pollutants due to their high toxicity to humans and other living organisms. They are also ubiquitous in our ecosystems as a result of industrial waste. Therefore, it is essential to develop a simple and inexpensive method to rapidly detect low concentrations of these metals in various liquids, including wastewater. Aptamer-based electrolyte-gated organic field-effect transistors (EGOFETs), which are traditionally used to detect large biomolecules such as viruses and proteins, are a promising concept for heavy-metal detection. To achieve this, we chose several aptamers with high affinity for and specificity to Pb^2+^ and Hg^2+^ ions, determined the dissociation constants and stoichiometry of the complexes, investigated the kinetics of complex formation and cross-specificity and proposed structures for the aptamer–cation complexes. Based on these aptamers, we developed EGOFET-based aptasensors for Pb^2+^ and Hg^2+^. The fabricated EGOFET devices have been shown to be sensitive to low concentrations of lead (from 30 nM to 2.5 μM) and mercury (from 250 nM to 2.5 μM) cations. These ranges correspond to the maximum permissible concentrations in drinking and wastewater for lead, which are 144 nM and 1.2 μM, respectively, and exceed the range of maximum permissible concentration in drinking and wastewater for mercury, which is 5 nM. Despite this, it was possible to detect mercury cations at a concentration of 5 nM within five minutes in a model system that simulates liquid flow using the EGOFET device. This demonstrates the potential for fast and selective detection of Pb^2+^ and Hg^2+^ ions at maximum permissible concentrations in drinking water using EGOFET-based aptasensors. This is a significant step forward in the development of flow sensors for detecting heavy metals in liquid media.

## 1. Introduction

Heavy metals are among the most hazardous environmental pollutants, with lead and mercury standing out due to their high toxicity and wide distribution in ecosystems [1,2]. Lead can accumulate in the human body and affect the nervous, hematopoietic, and renal systems [3]. Even at low concentrations, this metal can cause irreversible damage to cognitive development in children [4,5], while in adults, it may lead to progressive cardiovascular problems [6,7]. Mercury is also dangerous in various forms: elemental mercury is toxic to the respiratory system when inhaled [8], salts damage the kidneys [9,10], and metal–organic forms (e.g., methylmercury) easily cross the blood–brain barrier and cause serious damage to the central nervous system [11]. These metals can move through food chains and remain in the environment. Therefore, the long-term monitoring of these substances is a critical task for public health and environmental protection.

At the same time, traditional methods of lead and mercury detection, such as atomic absorption spectrometry and inductively coupled plasma mass spectrometry, require labor-intensive sample preparation, expensive equipment, and highly skilled personnel [12]. This makes continuous monitoring challenging, especially during the large-scale screening of drinking water, wastewater, or food samples outside laboratories.

In this context, point-of-care sensors with specific recognition elements can be used as an alternative to laboratory technologies. Sensors based on electrolyte-gated organic field-effect transistors (EGOFETs) with aptamers as recognition elements are traditionally used to detect large biomolecules such as viruses and proteins [13,14]. EGOFET-based aptasensors are also promising for detecting heavy metals. Nucleic acid aptamers are oligonucleotides with specificity to target analytes [15,16]. They have the advantage of being stable in various liquid media, easy to synthesize, and highly affine for a wide range of targets, including heavy-metal ions [17,18].

The mechanism of mercury recognition by aptamers is based on the specific interaction between Hg^2+^ ions and two thymine bases in single-stranded DNA. A mercury ion fits between two thymine bases, forming a stable T–Hg^2+^–T pair [19]. This pair differs from the canonical base pairs and causes a structural rearrangement in oligonucleotides. The high selectivity allows mercury to be distinguished from other cations with high reliability.

Lead recognition relies on a different structural motif. One of the key mechanisms is the stabilization of G-quadruplexes in the presence of Pb^2+^. Guanine-rich sequences can fold into four-stranded structures that are held together by planar guanine quartets. Lead ions effectively stabilize these conformations [20,21]. Other cations, like K^+^, Sr^2+^, and Na^+^, can also be coordinated within G-quadruplexes [22]. As a result, aptamers for Pb^2+^ are selective, but they are not absolutely specific. Their specificity to Pb^2+^ needs to be controlled, and their applicability is questionable in the presence of excessive amounts of other cations.

The key question in developing EGOFET-based aptasensors for detecting lead and mercury ions is whether aptamers can selectively bind to these ions not only in solution but also after they are immobilized on the surface of the EGOFET semiconducting layer.

In order to solve this problem, we selected several aptamers with high affinity for and specificity to Pb^2+^ and Hg^2+^ and investigated the kinetic and thermodynamic parameters of their complexes. Based on the selected aptamers, we constructed EGOFET-based aptasensors for Pb^2+^ and Hg^2+^. We used an optimized methodology for aptamer and analyte immobilization and measured sensitivity of the fabricated aptasensors to low concentrations (from 100 nM to 2.5 μM) of lead, mercury, and cadmium cations (control) in deionized water, drinking water, wastewater, and a model system that simulates liquid flow.

## 2. Results

### 2.1. Aptamer–Metal Cation Binding: Affinity and Specificity

The first step of this study involved the selection of aptamers that have high affinity for and specificity to Pb^2+^ and Hg^2+^. Several aptamers were chosen that bind to these metal ions based on previous studies [23,24,25,26,27]. The dissociation constants (K_D_) for some of these aptamer–metal complexes have been reported in the literature. For example, PbGQ has a K_D_ of 2.0 μM for its complex with Pb^2+^, as determined by isothermal titration calorimetry (ITC) [28]. Similarly, PbGQ2 has K_D_ values of 380 nM for its Pb^2+^ complex, as measured by circular dichroism titration [29], and 1.49 μM for its K^+^ complex, as determined by circular voltammetry (CVA) [30]. HD1 has K_D_ values of 96 nM for its Pb^2+^ complex, as determined by fluorescence anisotropy [31], and 1.13 μM for its K^+^ complex, as measured by CVA [30]. For aptamers binding to Hg^2+^ ions, their recognition site, the T-Hg^2+^-T complex, has a K_D_ of about 200 nM, as determined by isothermal titration calorimetry (ITC) [32].

**Figure 1:**
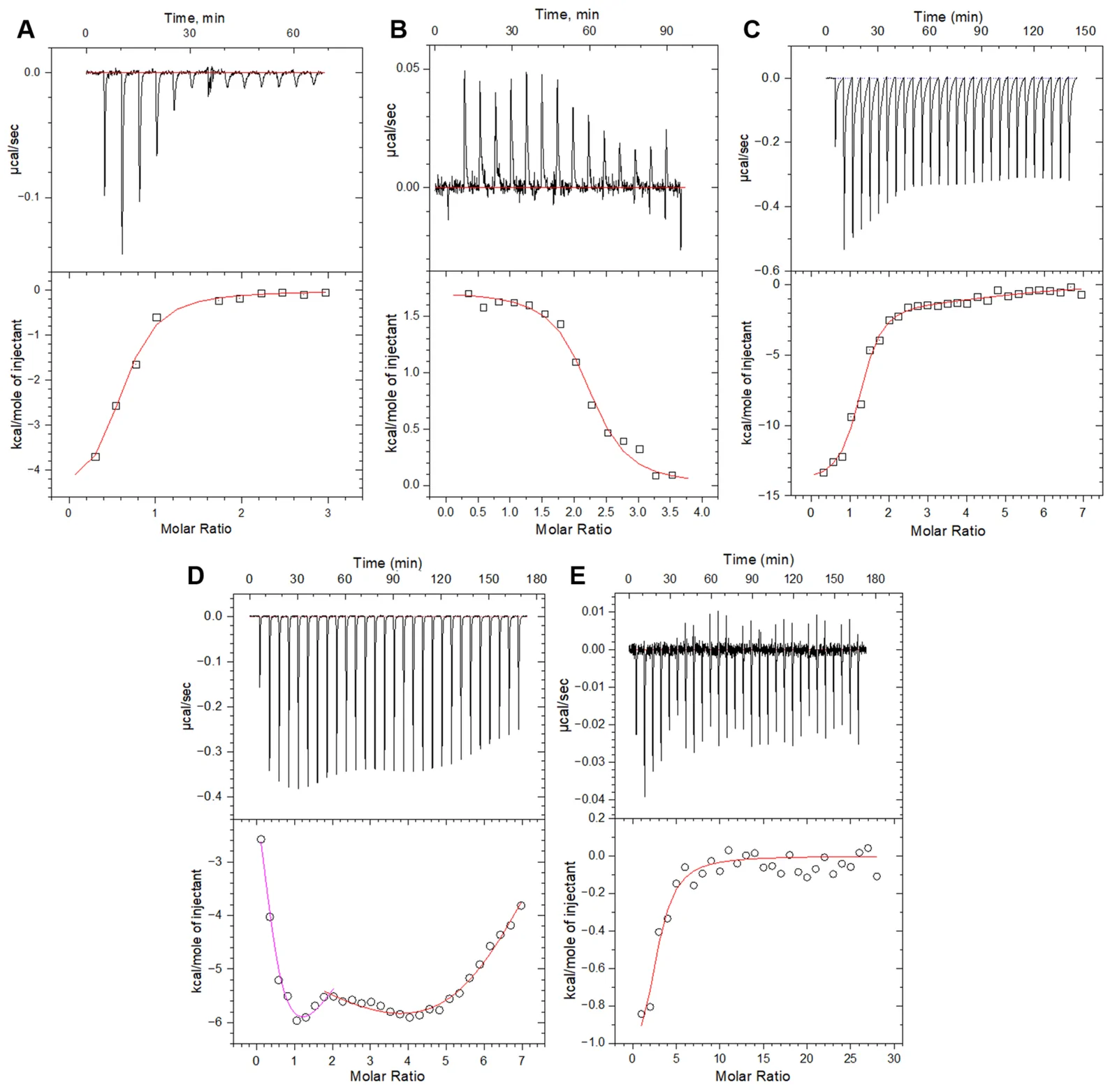
ITC spectra of 3 μM aptamer + 100 μM metal solution in 20 mM Tris-HNO_3_ buffer, pH 7.0, at 25 °C: (**A**) PbGQ + Pb(NO_3_)_2_, (**B**) PbGQ2 + Pb(NO_3_)_2_, (**C**) HD1 + Pb(NO_3_)_2_, (**D**) HgTT + HgCl_2_, and (**E**) HgTT2 + HgCl_2_.

First, we compared the affinity and specificity of the aforementioned aptamers under identical conditions. The DNA aptamers were modified with biotin, which is necessary for their subsequent immobilization on the EGOFET receptor layer. ITC was used to obtain a thermodynamic profile of the interaction, including the stoichiometry (N) and K_D_ values. The results confirmed the specific binding of the aptamers to the target ions, with K_D_ values ranging from tens to hundreds of nM (Table 1, Figure 1). In our experiments, the affinity for Pb^2+^ was even higher than in the literature; e.g., PbGQ had an-order-of-magnitude lower K_D_. However, K_D_ values of aptamer–Hg^2+^ complexes were two–three times higher than the literature values. During cation complexation, the aptamers showed different stoichiometries. One aptamer, HgTT, showed a complex behavior in at least two stages of complexation. These peculiarities can affect the robustness of heterogeneous EGOFET-based sensors. Therefore, it is interesting to compare aptamers with different mechanisms.

It is widely known that the formation of G-quadruplexes is highly dependent on the presence of metal cations, especially Na^+^ and K^+^ [33]. Since the aptamers chosen for Pb^2+^ have a G-quadruplex structure, it was necessary to analyze the influence of Na^+^ or K^+^ on their ability to bind to the target analyte. For this purpose, an ITC experiment was performed in a buffer with a high concentration of sodium ions (140 mM NaNO_3_). A decrease in the heat effect upon binding the aptamers PbGQ and PbGQ2 to Pb^2+^ ions was observed (see Supplementary Materials, Figure S1). This can be explained by the competition between Pb^2+^ and Na^+^ for the active binding sites on the aptamer. In contrast, the aptamers for Hg^2+^ showed signal stability in the presence of sodium, suggesting the absence of competitive inhibition and the potential for use in saline environments.

A study on the cross-specificity of the aptamers PbGQ2 and HgTT2 to cadmium ions (Cd^2+^) with a size comparable to that of the target cations yielded the following results (Table 1, Supplementary Materials, Figure S2): The PbGQ2 aptamer has low cross-specificity to cadmium, with K_D_ values differing by two orders of magnitude. In contrast, the HgTT2 aptamer displays distinct cross-specificity, with K_D_ values for the complexes with the target cation and the cadmium cation differing by only a factor of two.

Stopped-flow kinetics in fluorescent spectroscopy mode was used to monitor the kinetics of the assembly of the aptamer–metal complexes, yielding a characteristic timeline of the interactions. Also, cross-specificity to several other divalent cations was tested. Based on their high affinity for target cations and applicability in previous studies, we selected PbGQ and HgTT for detailed investigation. The interactions between target and off-target metal ions with aptamers labeled with a fluorescein tag (FAM) at different positions (PbGQl, PbGQe, HgTT3, and HgTT6) were investigated.

**Figure 2:**
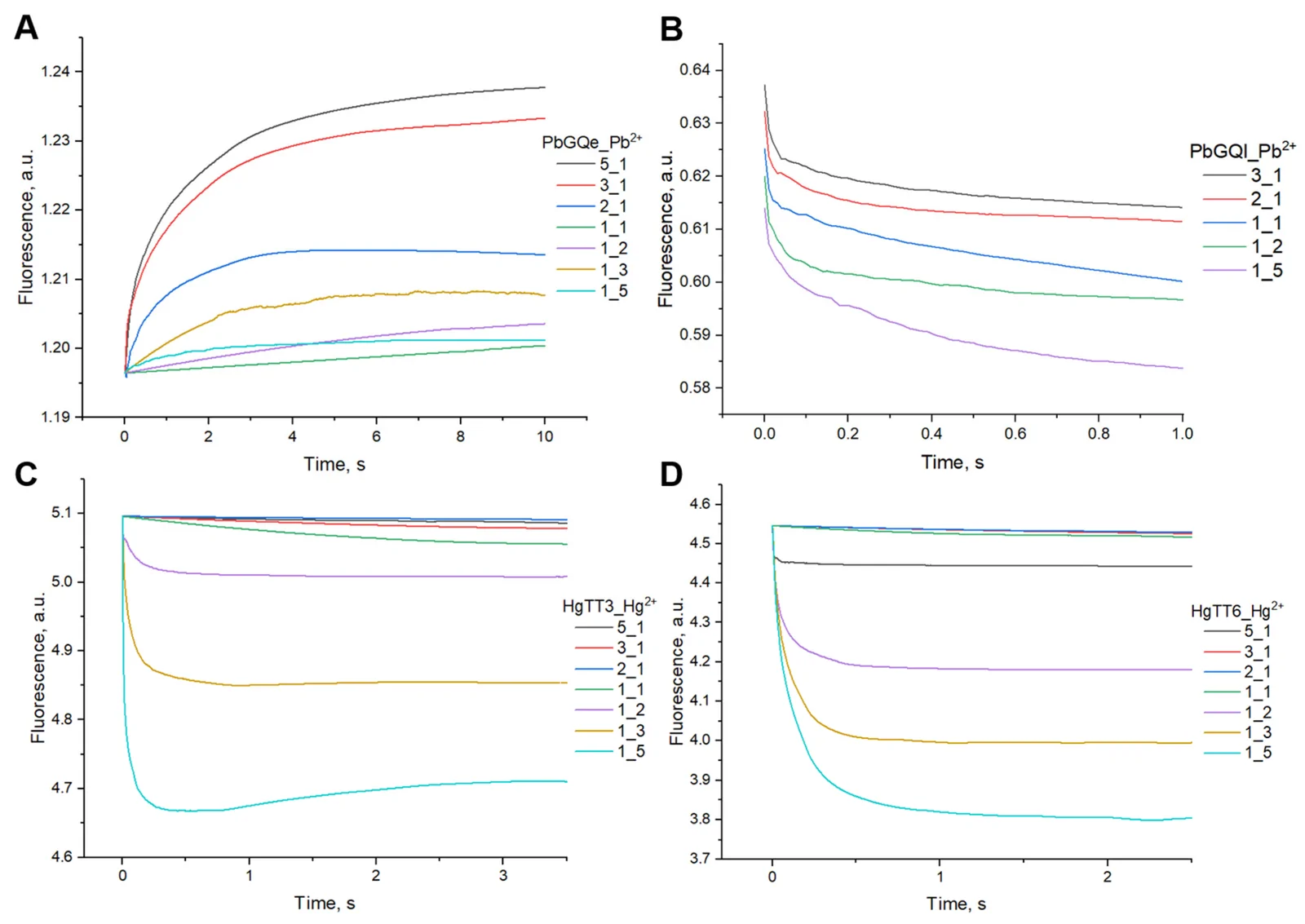
Stopped-flow kinetic experiments. Kinetics of (**A**) PbGQe and (**B**) PbGQl aptamers in complexation with Pb^2+^ and kinetics of (**C**) HgTT3 and (**D**) HgTT6 aptamers in complexation with Hg^2+^.

Since it has previously been assumed that the PbGQ aptamer dimerizes during Pb^2+^ binding, the FAM tag was positioned at the 5′ terminus (PbGQe) and the fourth nucleotide located in the G-quadruplex loop (PbGQl). The rapid increase in fluorescence observed for the end-labeled variant of the PbGQe aptamer reflects the stacking-out of the tag during dimerization (Figure 2). At the same time, the rapid decrease in fluorescence of the PbGQl aptamer indicates the trapping of the FAM into the G-quadruplex groove, which supports Pb^2+^-induced G-quadruplex folding. The calculated affinity was even higher compared to ITC data: the K_D_ calculated from fluorescence measurements was equal to 34 nM (Table 2), while the K_D_ of 270 nM was calculated from ITC data. Differences in the calculated affinity can be attributed to aptamer modification with FAM. The stoichiometry was two aptamers per Pb^2+^ ion for PbGQe, in full agreement with ITC data, whereas PbGQl showed a 1:1 stoichiometry. The differences between PbGQe and PbGQl are related to FAM localization. When FAM is in the loop, it is not affected by dimerization, but when it is at the end, it undergoes significant changes in its environment during dimerization. As a result, the data for PbGQe are more reliable than those for PbGQl. Similar differences between fluorescence changes and label localization have been described previously for G-quadruplex and i-motif structures [34,35]. Fluorescence changes should be interpreted with caution, and a thorough comparison of several labeled variants, as well as additional techniques like ITC, should be used.

Regarding mercury, which is recognized by aptamers through T-Hg^2+^-T base pairing, the kinetics of the aptamer HgTT were examined using modifications of the third and sixth thymine residues with the FAM tag. The resulting fluorescence quenching in both cases may indicate the stacking of FAM with a newly folded structure. Most probably, these curves indicate aptamer dimerization with the formation of a duplex with mismatched TT pairs. The affinity for Hg^2+^ and cross-specificity depended on the site of modification. HgTT6 had the closest K_D_ to ITC data (350 nM vs. 350 nM; Table 2), while HgTT3 has three times lower affinity, along with the lack of specificity to Hg^2+^/Cd^2+^. These results may indicate that the FAM in the third position interferes with the binding site, obstructing aptamer performance. HgTT3 also shows signs of the second step in complexation. Modification in the sixth position of HgTT6 provided a K_D_ value similar to that of ITC, and this variant was chosen as more reliable compared to HgTT3. Compared to HgTT2, HgTT had low cross-specificity to control ions, which is beneficial for biosensor performance.

In all cases studied, complexation was completed within a few seconds. This rapid complexation is consistent with the accumulation regime of biosensing using point-of-care instruments for environmental measurements of cation content.

### 2.2. Structure of Aptamer–Metal Complexes and Complex Stability Characterization

Based on the stoichiometry of aptamer–metal complexes derived from ITC and stopped-flow kinetic data, as well as the unique features of aptamer sequences, we proposed potential structural models of these complexes. The DNA topologies were subsequently validated using circular dichroism (CD) spectroscopy.

**Figure 3:**
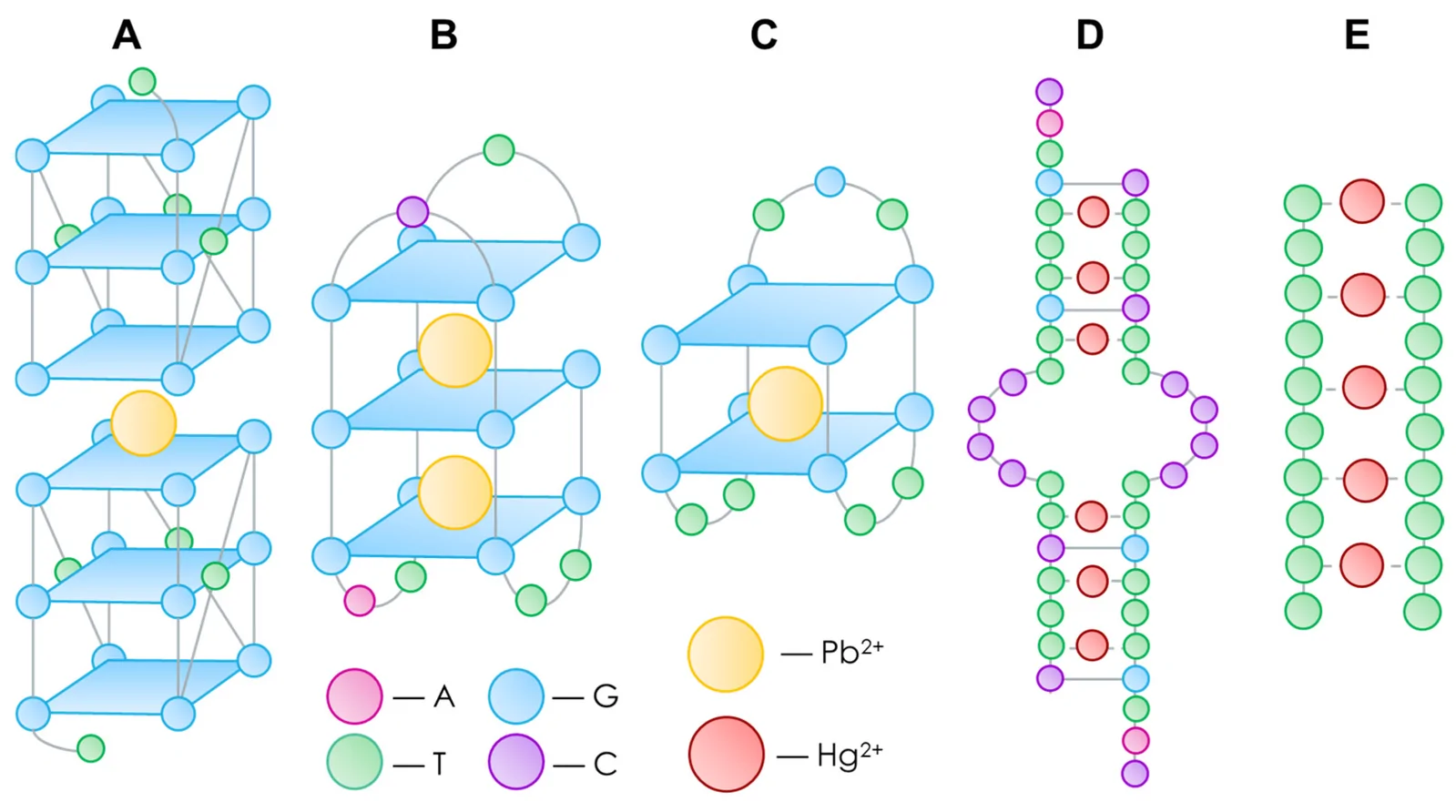
Proposed structures of the aptamer complexes with target cations: (**A**) PbGQ, (**B**) PbGQ2, (**C**) HD1, (**D**) HgTT, and (**E**) HgTT2.

**Figure 4:**
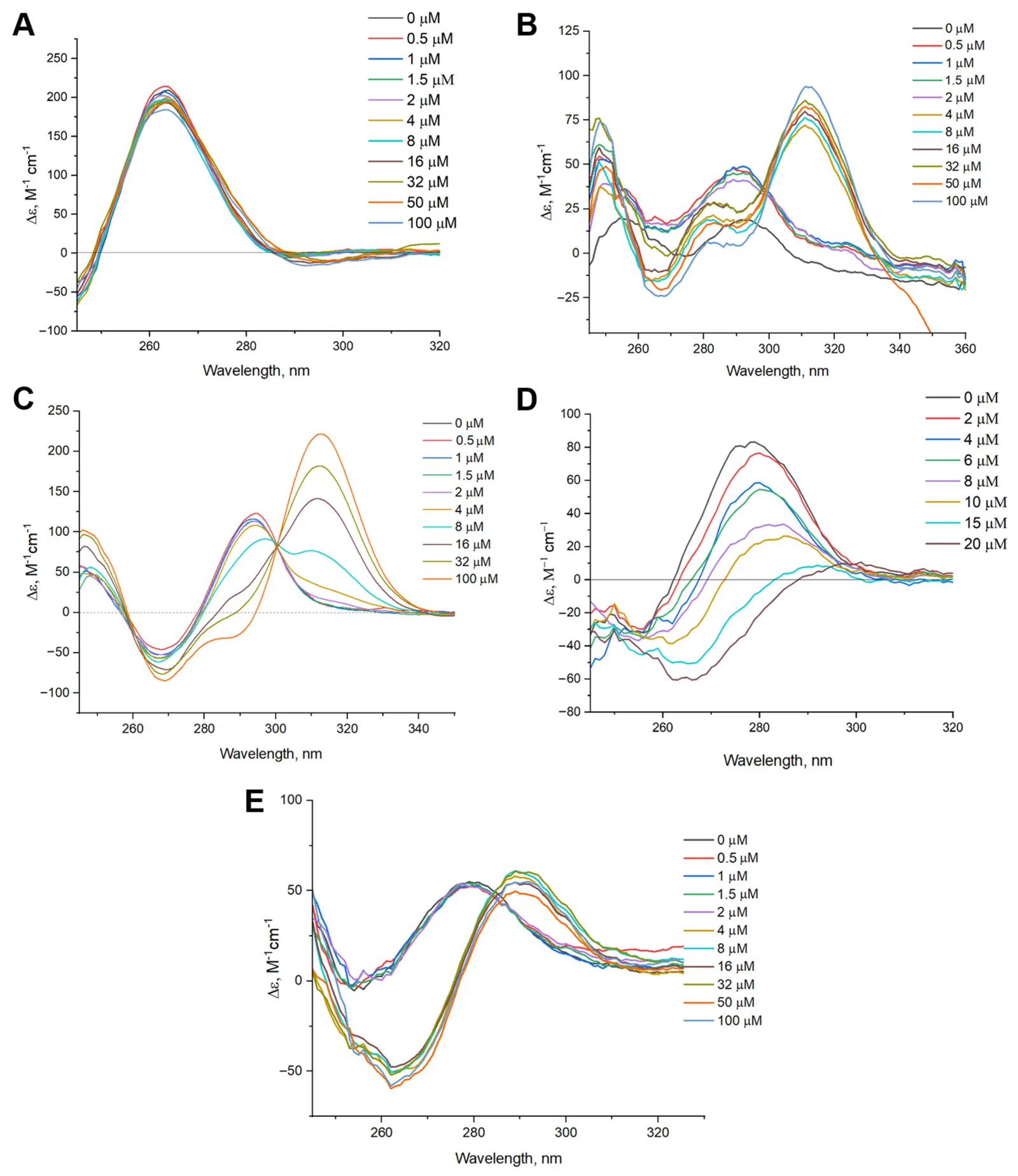
CD spectra of 2 μM of different aptamers with Pb^2+^ (**A**–**C**) and Hg^2+^ (**D**,**E**) cations with concentrations in the range of 0–100 μM: (**A**) PbGQ; (**B**) PbGQ2; (**C**) HD1; (**D**) HgTT; (**E**) HgTT2.

Affinity analysis revealed a 2:1 stoichiometry between the PbGQ aptamer and lead cations. Given the presence of G-repeats, typical of G-quadruplexes, it was proposed that the aptamer forms a dimer when binding to Pb^2+^, with another face of the parallel G-quadruplex remaining hidden by terminal nucleotides (Figure 3A). Similar G-quadruplex dimers were described earlier [36]. This parallel G-quadruplex architecture was confirmed through CD spectroscopy, which showed a prominent peak at 260 nm, characteristic of parallel G-quadruplexes. Additionally, the minor spectral shifts upon the addition of Pb^2+^ suggested that the metal interacts with an already formed G-quadruplex rather than inducing dimerization (Figure 4A).

CD spectroscopy was also used to evaluate the thermal stability of the resulting complexes. For the PbGQ aptamer, these measurements were conducted with a 10:1 ratio of Pb^2+^ ions (10 µM) to the aptamer concentration. The melting temperature of the complex was 72.6 ± 1.9 °C, which is 6 °C higher than that of the aptamer in the absence of Pb^2+^ cations (66.9 ± 1.2 °C) (Supplementary Materials, Figure S3). Therefore, Pb^2+^ ions stabilize the preformed dimeric structure of PbGQ, and we did not expect significant conformational changes in this aptamer during EGOFET-based sensor operation.

The PbGQ2 aptamer, which also showed binding affinity for Pb^2+^ ions, was found to interact with the metal in a 1:2 aptamer:metal molar ratio. Based on these results and the oligonucleotide sequence, we propose that PbGQ2 forms an antiparallel G-quadruplex that intercalates two metal ions, which correlates well with the structure described previously [37]. CD spectroscopy confirmed that PbGQ2 folds into an antiparallel G-quadruplex in the absence of Pb^2+^. This is evidenced by a characteristic peak at 295 nm (Figure 4B). When Pb^2+^ concentrations increase, the spectrum shifts to 310 nm, indicating the formation of antiparallel G-quadruplex complexes with divalent metal cations [38]. Furthermore, thermal stability assays showed that the aptamer is inherently unstable (T_m_ = 28 ± 2 °C) but becomes significantly more stable when excess Pb^2+^ is added, resulting in a melting temperature of 72.4 ± 1.1 °C (Supplementary Materials, Figure S4).

We also tested the thrombin-binding HD1 aptamer, which is capable of forming a 1:1 complex with Pb^2+^, as shown by ITC. Similar to PbGQ2, the free aptamer forms an antiparallel G-quadruplex, as evidenced by a peak at 295 nm that shifts to 310 nm upon binding to Pb^2+^ ions (Figure 4C). Additionally, CD spectroscopy in “melting” mode showed that the interaction with Pb^2+^ significantly increases the thermal stability of HD1. The melting temperature rose from 29.3 ± 0.2 °C in the absence of cations to 60.5 ± 0.3 °C in their presence (Supplementary Materials, Figure S5). A possible structure of the complex is shown in Figure 3.

As for the aptamers for mercury cations, ITC and stopped-flow kinetic data analysis revealed a stoichiometry of two HgTT aptamers per Hg^2+^ ion, with subsequent saturation of the aptamers with three additional Hg^2+^ ions per oligonucleotide. CD spectroscopy showed that the aptamer lacks a secondary structure in the absence of mercury. When mercury is present, the CD spectrum changes significantly, indicating complex formation (Figure 4D). The melting temperature of the complex was found to be 40 °C (Supplementary Materials, Figure S6). A possible structure for the complex is shown in Figure 3.

The HgTT2 aptamer was characterized to bind three cations per aptamer. The DNA hairpin, composed of 10 thymine residues, is very unstable. Therefore, the most possible structure includes a DNA dimer that binds to 5 Hg^2+^ ions (Figure 3). Similar to HgTT, the HgTT2 aptamer showed clear CD spectral changes upon addition of mercury. Interestingly, the HgTT2 complex with Hg^2+^ exhibited high thermal stability, as its CD spectra remained stable across temperature gradients up to 90 °C (Supplementary Materials, Figure S7).

Additionally, CD spectroscopy was used to assess the effect of FAM labeling on the aptamers’ properties. In the absence of cations, PbGQl and PbGQe showed a decrease in thermal stability, with melting temperatures of 56.8 ± 0.9 °C and 57.7 ± 0.7 °C, respectively, compared to the unlabeled variant with a melting point of 66.9 ± 1.2 °C. Conversely, when Pb^2+^ was added, both labeled aptamers showed increased stability, reaching melting temperatures of 78.9 ± 0.8 °C and 78.5 ± 0.7 °C, respectively, which are higher than the 72.6 ± 1.9 °C for the unlabeled sequence (Supplementary Materials, Figures S8 and S9). The overall structure of the aptamers remained the same, but their thermal stability changed by 5–10 °C. With regard to mercury detection, the labeled variant of HgTT3 exhibited improved sensitivity, as evidenced by a shift in the peak at 4 μM Hg^2+^. The unlabeled HgTT required 8 μM to achieve the same result, while HgTT6 is more similar to the unlabeled variant (Supplementary Materials, Figure S10). These data indicate that the modified aptamer variants retain the same structural features, with some differences in detail.

### 2.3. The Architecture of the EGOFET-Based Sensor and Recognition-Layer Formation

#### 2.3.1. Optimization of Recognition Layer: Aptamer and Streptavidin Concentrations

**Figure 5:**
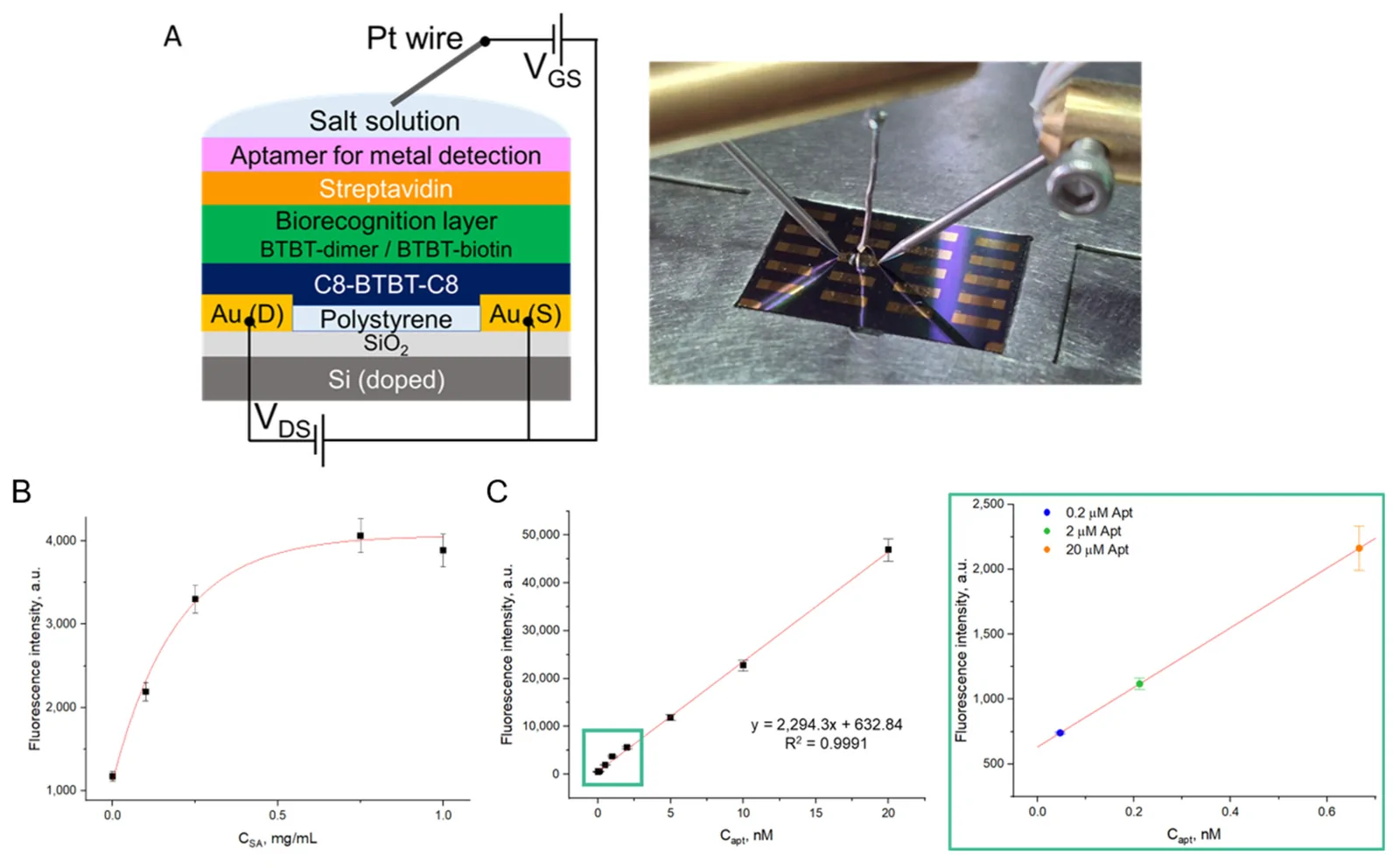
EGOFET-based sensor architecture and photo of a 20-pixel chip measurement (**A**). Optimization of streptavidin concentration (**B**). Calibration curve of fluorescently labeled oligonucleotide: dependence of fluorescence of the washes on oligonucleotide concentration during immobilization procedure (**C**).

The second step of this study focused on the development of EGOFET sensors based on selected aptamers and the evaluation of their performance. Our previous research explored the use of aptamers as recognition elements in EGOFET-based sensors for detecting influenza A virus. Those studies resulted in device architectures with different configurations and detection limits of 6.1∙10^4^ VP/mL [39] and 8∙10^4^ VP/mL [40]. Additionally, we developed a multisensory platform using an aptamer-based pH-dependent switch that could detect influenza A virus and Ag^+^ cations at different pH [40]. Based on insights gained from our previous investigation into EGOFETs and the models of aptamer–cation complexes, we proposed that the sensor configuration described in reference [41] (Figure 5A) could effectively detect mercury and lead cations in aqueous media.

Considering the significant differences in particle size of the influenza A virus and cations, as well as the dimerization in some aptamer–metal complexes, it was necessary to determine the optimum contents of streptavidin and aptamer on the surface of the EGOFET semiconducting layer. To identify the optimal streptavidin density for maximum sensor performance, EGOFET surfaces were functionalized with streptavidin at concentrations ranging from 0.1 to 1 mg/mL. Afterwards, oligonucleotides labeled with a fluorescent tag were immobilized on the functionalized surface. The receptor layer consisting of streptavidin and the labeled oligonucleotide was then recovered from the EGOFET surface using DMSO, and the resulting fluorescence of the solution was analyzed to assess binding. Fluorescence was linearly dependent on the concentration of the oligonucleotide. Saturation of the response was achieved at a streptavidin concentration of more than 0.5 mg/mL (Figure 5B), and this concentration was therefore accepted as optimal. In our previous studies, we used a lower streptavidin concentration of 0.1 mg/mL, so it was necessary to check whether a higher concentration would negatively affect the electrical properties of the EGOFETs. We found that a dense layer of immobilized streptavidin on the EGOFET surface did not degrade device performance (Supplementary Materials, Figure S11). The transfer characteristics of the EGOFET devices with a top streptavidin layer, measured in 20 devices on the same substrate, also demonstrated excellent device-to-device reproducibility.

To determine the efficiency of aptamer immobilization on the EGOFET surface, a biotinylated oligonucleotide with a fluorescent tag was immobilized on the sensor surface with the top streptavidin layer at concentrations ranging from 0.2 μM to 20 μM. Then, the labeled oligonucleotide was recovered from the EGOFET surface using dimethyl sulfoxide (DMSO); and the fluorescence of the resulting solution was analyzed using the calibration curve (Table 3, Figure 5C, Supplementary Materials, Figure S12).

The increase in oligonucleotide solution concentration during immobilization led to an increase in the amount of the immobilized oligonucleotide. Calculations showed that the surface density of the immobilized oligonucleotide reaches 20% of the theoretical maximum for a fully occupied monolayer, resulting in the retention of nanomolar amounts of the oligonucleotide on the EGOFET surface. The observed difference between the high concentrations (μM) of solutions used for oligonucleotide immobilization and the low concentrations (nM) of oligonucleotide solutions washed off the EGOFET surface suggests that many binding sites on streptavidin are sterically hindered, requiring a significant molar excess of the oligonucleotide to effectively functionalize the surface.

**Figure 6:**
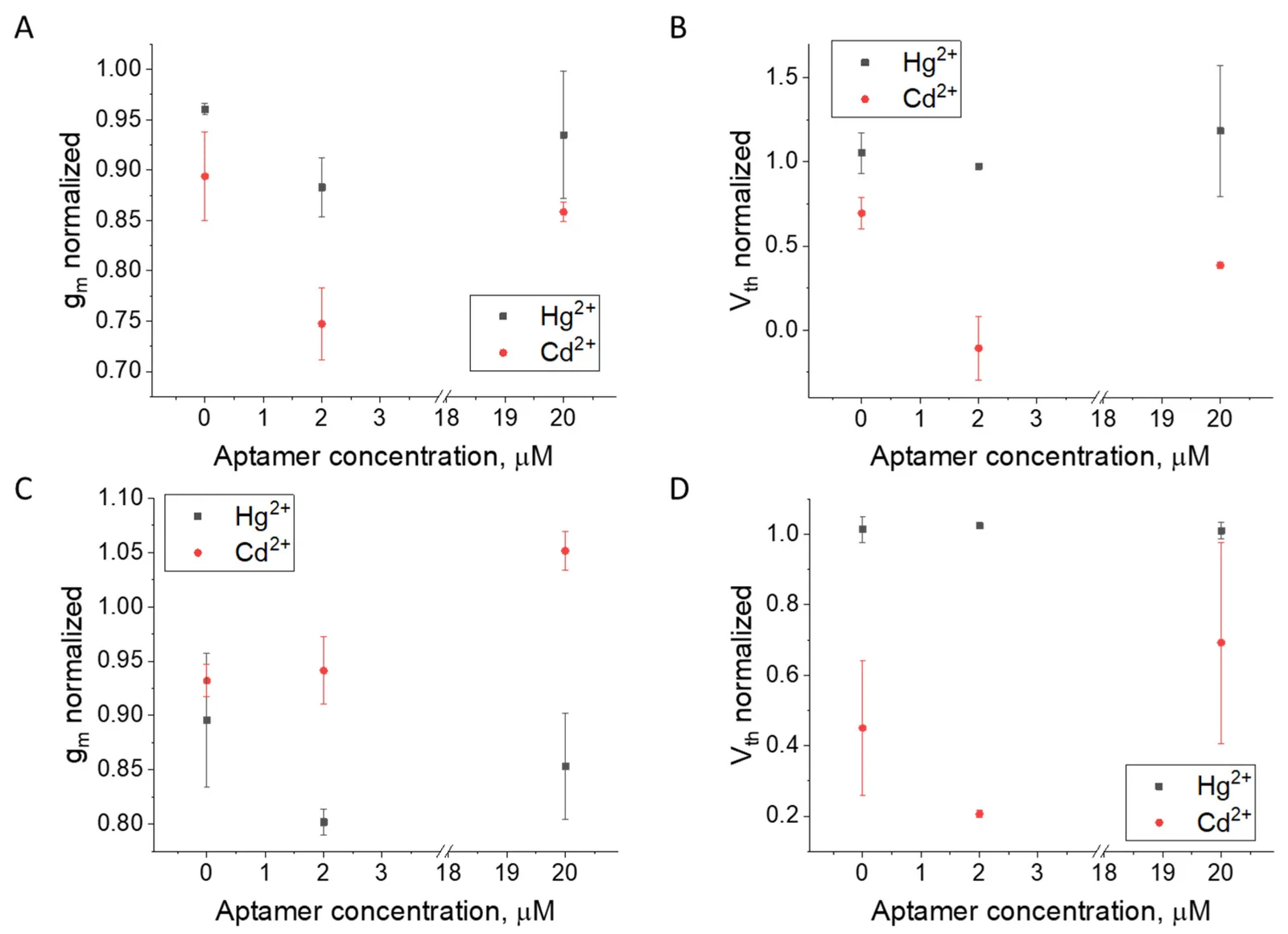
The responses of the EGOFET devices to 4 μM target mercury and control cadmium cations for the aptamers HgTT (**A**,**B**) and HgTT2 (**C**,**D**) at concentrations of 0 ÷ 20 µM based on the sensor response parameters g_m normalized_ (**A**,**C**) and Vth _normalized_ (**B**,**D**).

**Figure 7:**
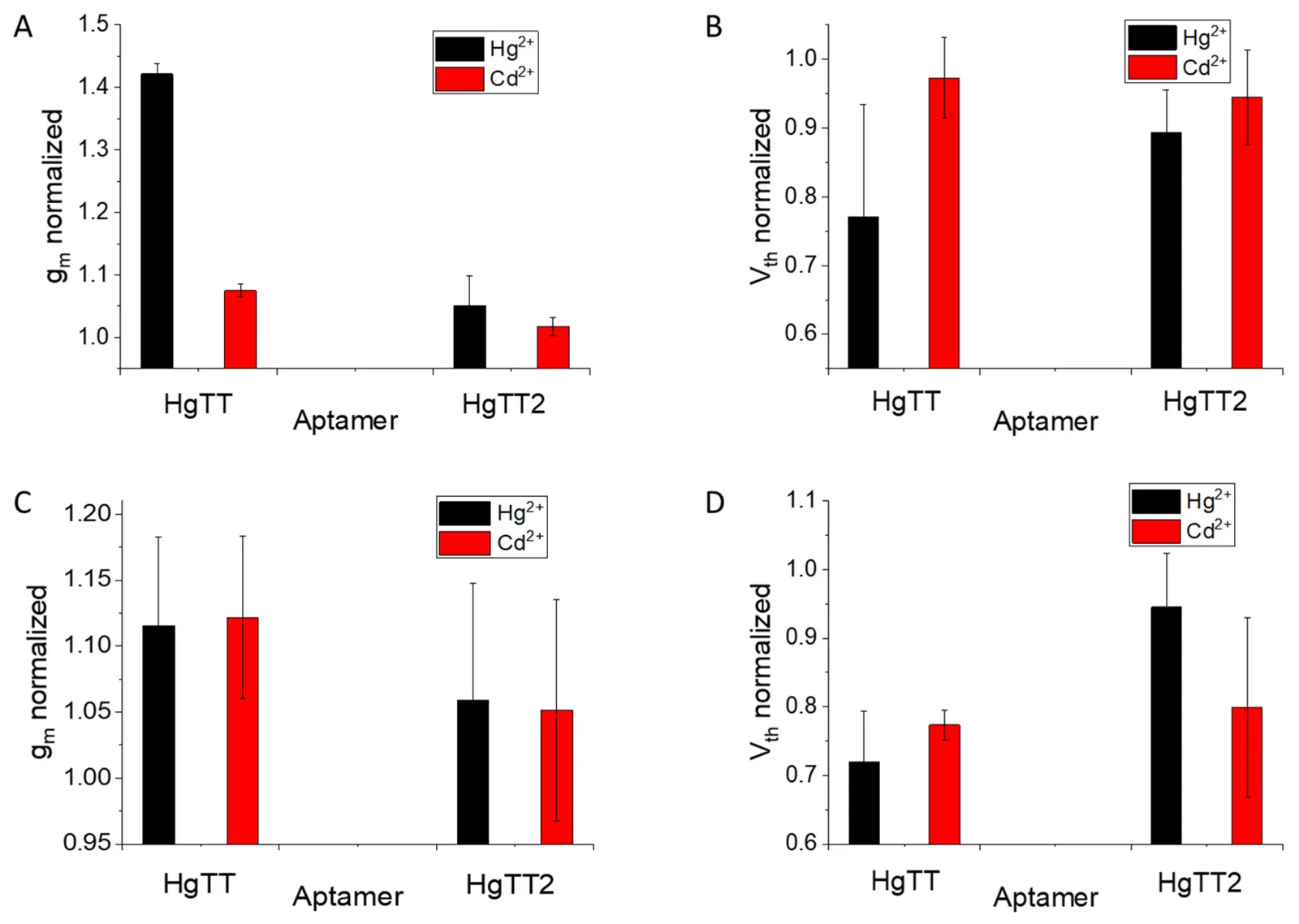
The responses of the EGOFET devices—g_m normalized_ (**A**,**C**) and Vth _normalized_ (**B**,**D**)—to target (Hg^2+^) and control (Cd^2+^) cations immobilized using two incubation techniques—joint (**A**,**B**) and consecutive (**C**,**D**)—for HgTT and HgTT2 aptamers.

**Figure 8:**
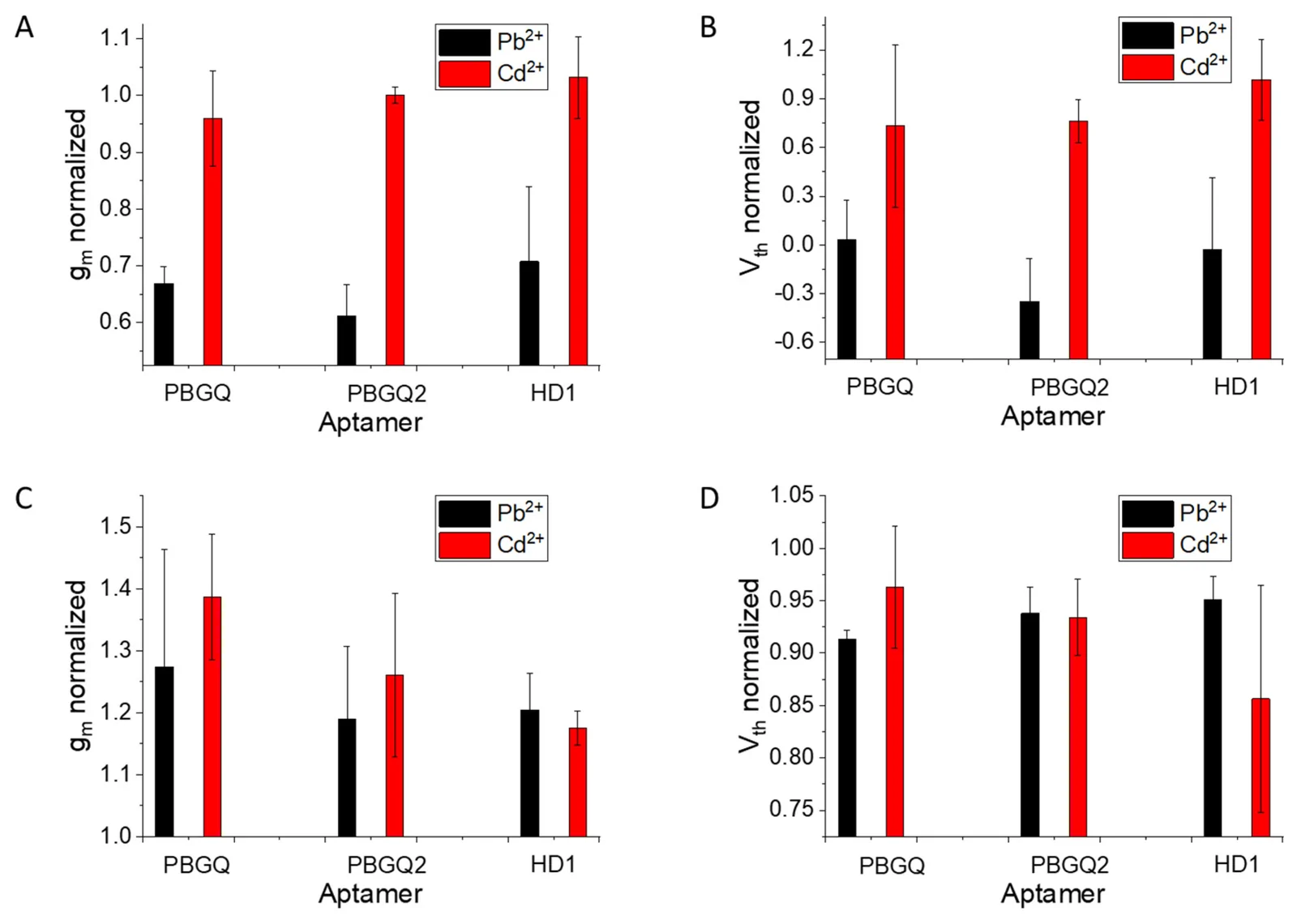
The responses of the EGOFET devices—g_m normalized_ (**A**,**C**) and Vth _normalized_ (**B**,**D**)—to target (Pb^2+^) and control (Cd^2+^) cations immobilized using two incubation techniques—consecutive (**A**,**B**) and joint (**C**,**D**)—for PbGQ, HD1 and PbGQ2 aptamers.

**Figure 9:**
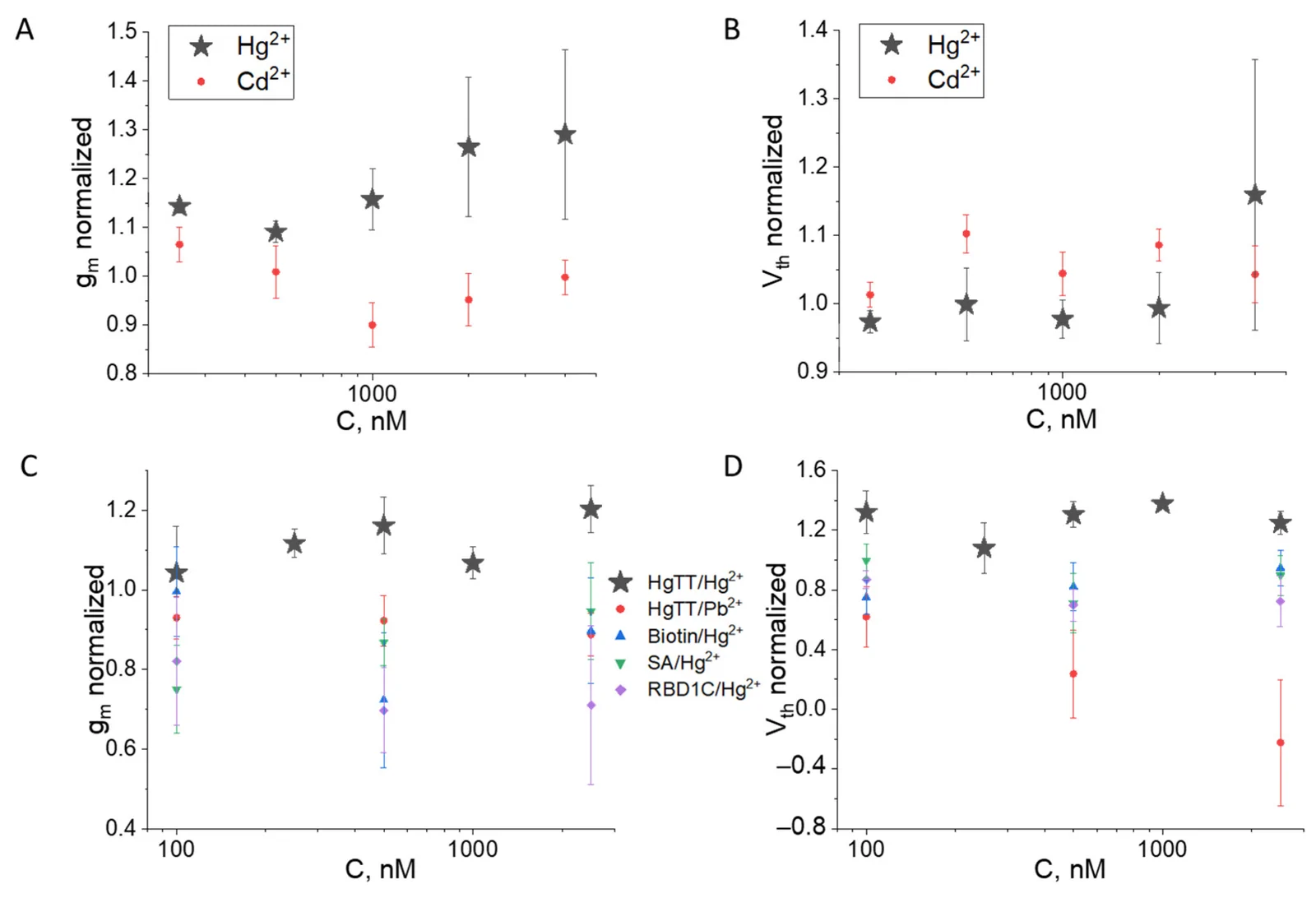
The dependence of the responses of EGOFET sensors on the concentrations of the target Hg^2+^ and control Cd^2+^ (**A**,**B**) and Pb^2+^ (**C**,**D**) cations in terms of g_m normalized_ (**A**,**C**) and Vth _normalized_ (**B**,**D**) measured for the different EGOFET top layers: biotinylated (biotin; blue points), streptavidin-coated (SA; green points) and modified with the aptamer specific to Hg^2+^ (HgTT; black and red points), as well as the aptamer (RBD1; violet points) specific to SARS-CoV-2 virus and non-specific to metal cations.

In the next step, we determined the optimal concentration of the aptamer to achieve the best selectivity of the EGOFET sensor. It was demonstrated that the selectivity of the sensor for target mercury cations, compared to control cadmium cations, depends on the concentration of aptamer solution used during the surface functionalization process. The selectivity of sensors for mercury cations decreased with the increase in concentrations of both aptamer solutions (HgTT and HgTT2). This was evidenced by the gradual convergence of sensor responses to mercury and cadmium cations at a concentration of 4 µM for both sensor response parameters—normalized threshold voltage (Vth) and transconductance (g_m_) (Figure 6). The normalized responses of the electrical parameters of the EGOFETs (g_m normalized_ and Vth _normalized_) were calculated using the formula X_apt+Me_/X_SA_, where X_apt+Me_ is the electrical parameter of the EGOFET with the aptamer–metal complex on the top layer and X_SA_ is the electrical parameter of the EGOFET with the streptavidin layer on top. These normalized parameters were also used for the graphs in Figure 7, Figure 8 and Figure 9.

For Hg^2+^ detection, the optimal concentration of the HgTT aptamer was found to be 2 μM, while the optimal range for HgTT2 aptamer concentration was established as 2–20 μM, depending on the sensor response parameter used (Figure 6). In contrast, in experiments with EGOFET surfaces modified with labeled oligonucleotides, the best sensor selectivity was achieved at an aptamer concentration of 2 μM. This difference can be attributed to some steric hindrance caused by the complex assembly on the sensor surface after joint incubation with metal cations (see Section 2.3.2).

The semiconducting and biotinylated receptor layers of the EGOFET sensor were characterized using polarizing optical microscopy (POM) (see Supplementary Materials, Figure S13A,B). The thinner layers, including the streptavidin, PbGQ, and HD1 aptamer layers, were characterized using atomic force microscopy (see Supplementary Materials, Figure S13C–F). For each stage of modification, images that were typical of a successful step were obtained, as previously described [39,40,41,42].

#### 2.3.2. Selection of a Metal Cation Detection Technique

Taking into account the dimerization in several aptamer–metal complexes, which may lead to difficulties in the formation of these complexes directly on the EGOFET surface, two approaches for target cation detection were compared: (1) incubation of the target cations on the sensor surface modified with the aptamers (consecutive incubation) and (2) joint incubation of the aptamers and target cations in a solution, followed by immobilization of the resulting complex on the surface (joint incubation). Experiments were conducted to examine the effects of these two detection techniques, consecutive and joint incubation, using two aptamers (HgTT and HgTT2) and target (Hg^2+^) and control (Cd^2+^) cations at a concentration of 2 µM. The EGOFET sensor responses were calculated in terms of normalized electrical parameters (g_m normalized_ and Vth _normalized_) according to the following formulae: X_apt/Me_/X_SA_ for the consecutive technique or X_apt+Me_/X_SA_ for the joint technique, where X_apt/Me_ is the electrical parameter of the EGOFET with a top layer with immobilized cations obtained using the consecutive technique, X_apt+Me_ is the electrical parameter of the EGOFET with a top layer with immobilized aptamer–metal complexes obtained by the joint technique, and X_SA_ is the electrical parameter of the EGOFET with a top streptavidin layer. Both signal acquisition and analysis revealed significant differences in data distribution, as well as the importance of the incubation approach for sensor selectivity for target and control cations. As shown in Figure 7, the joint incubation technique provided better separation between device responses to target (Hg^2+^) and control (Cd^2+^) ions compared to consecutive incubation. However, in both cases, one can choose a sensor response parameter (e.g., g_m_ for joint incubation or Vth for consecutive incubation) that can be used to separate target and control cations. The joint technique proved to be universal in terms of the parameters that can be used to distinguish between target and control cations. For example, for transconductance (g_m_) in the case of the HgTT aptamer, the sensor responses to Hg^2+^ and Cd^2+^ differed by approximately 5-fold, with the lowest dispersion of normalized g_m_ values measured for several EGOFET pixels, and, accordingly, with the lowest error. EGOFET devices based on the HgTT2 aptamer demonstrated lower stability and selectivity, as was also observed in subsequent experiments on sensor sensitivity estimation.

The same experiment was carried out for EGOFET devices based on three aptamers specific to Pb^2+^ cations at a concentration of 2.5 µM (Figure 8). Compared to mercury detection, the consecutive technique yielded better results in distinguishing the target (Pb^2+^) from the control (Cd^2+^) cations for lead detection. Both the normalized g_m_ and Vth response parameters were informative for the consecutive technique, but only the Vth parameter can be used with lower efficiency to distinguish lead and cadmium in the joint incubation technique. This trend was maintained for all three aptamers, indicating a specific interaction between lead and the EGOFET layers.

### 2.4. Calibration Curves for the EGOFET Aptasensors for Mercury and Lead

For further experiments, we selected the PbGQ2 and HgTT aptamers, as they showed 1000-fold differences in affinity between target and control cations (Table 2), as well as better selectivity for target and control cations in EGOFETs compared to other aptamers. In the case of mercury detection, transconductance was proven to be the best response parameter, due to the better separation between the observed responses to target (Hg^2+^) and control (Cd^2+^ and Pb^2+^) ions (Figure 9 and Figure S15). In both cases, the normalized transconductance increased with the increase in mercury concentration and decreased with the increase in cadmium and lead concentrations. This indicates that the EGOFETs’ electrical performance improved in the presence of mercury and degraded in the presence of cadmium and lead. The blank variability was calculated as the response of the EGOFETs with an aptamer-coated surface to deionized water according to the formula: g_m Apt_/g_m Apt AVG_, where g_m Apt_ is the transconductance of the EGOFET with the aptamer-coated surface measured in a water drop and g_m Apt AVG_ is the substrate-averaged transconductance of the EGOFET with aptamer-coated surface also measured in a water drop. The blank variability for the HgTT aptamer, specific to Hg^2+^, was found to be 1.00± 0.03. Using this blank variability and a regression equation (see Supplementary Materials, Table S1), the limit of detection (LoD) of 250 nM and the limit of quantification (LoQ) of 1000 nM were calculated for Hg^2+^ detection as 3δ and 6δ deviations, respectively, of the blank response.

**Figure 10:**
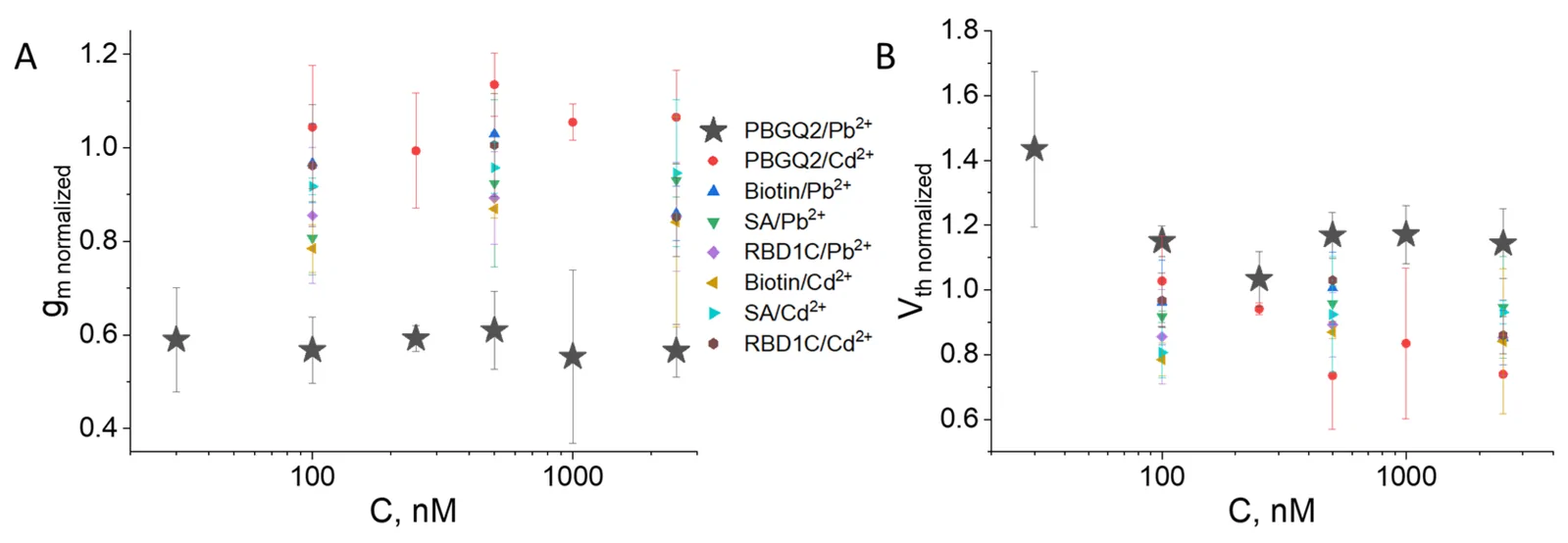
The dependence of the EGOFET sensor response on the concentration of target Pb^2+^ and control Cd^2+^ cations: g_m normalized_ (**A**) and Vth _normalized_ (**B**) measured for the different EGOFET surfaces, including biotinylated (biotin; blue and beige points), streptavidin-coated (SA; green and light blue points) and modified with the aptamer specific to Pb^2+^ (PbGQ2; black and red points) and the non-specific aptamer (RBD-1C; violet and brown points).

The calibration curves for the EGOFETs based on the PbGQ2 aptamer are provided in Figure 10 and Figure S15. As with mercury detection, transconductance was found to be the best response parameter, due to the better separation of the response to target (Pb^2+^) and control (Cd^2+^) ions (Figure 10A). The EGOFET sensors’ response parameters (g_m normalized_ and Vth _normalized_) were calculated using the formula X_apt+Me_/X_apt_, where X_apt+Me_ is the parameter of the EGOFET with aptamer–metal complexes on the top layer and X_apt_ is the parameter of the EGOFET with aptamers on the top layer. This approach to normalizing response parameters allowed us to take into account only changes associated with the immobilization of metal cations on the EGOFET surface while eliminating the influence of the underlying functional layers on the electrical properties of the EGOFET. As can be seen from the calibration curves (Figure 10), transconductance did not change significantly in the presence of the control cadmium cations. The normalized transconductance value was about 1.0 for a wide range of Cd^2+^ concentrations. In contrast, the presence of lead cations caused the transconductance to decrease by almost 2 times (down to 0.55 g_m. apt_) even at a Pb^2+^ concentration of 100 nM. It is worth noting that the response of transconductance to Pb^2+^ appeared to plateau across the tested concentrations (Figure 10A). The blank variability for devices with the PBGQ2 aptamer was 1.00 ± 0.08, indicating that all responses to Pb^2+^ were beyond the blank variability of the device, calculated as 3δ deviations. The normalized threshold voltage can be used as a parameter of the sensing lead cations in the concentration range from 30 nM to 250 nM. At higher concentrations, the response became saturated. The blank variability for normalized threshold voltage was equal to 1.00 ± 0.05. Based on this blank variability and the regression equation (see Supplementary Materials, Table S1), the lead LoD was calculated to be 30 nM, which corresponds to 3δ deviations from the blank response.

In order to investigate the non-specific interactions between EGOFET layers and metal cations, additional experiments were performed. Control experiments were conducted for both target (Pb^2+^ and Hg^2+^) and control (Cd^2+^ and Pb^2+^, respectively) ions made to interact with different EGOFET surfaces: biotinylated (biotin), streptavidin-coated (SA) and modified with the RBD-1C aptamer specific to SARS-CoV-2 virus and non-specific to metal cations (Figure 9 and Figure 10). For the control experiments, EGOFET sensor response parameters (g_m normalized_ and Vth _normalized_) were calculated using the formula: X_layer+Me_/X_layer_, where X_layer+Me_ is the parameter of the EGOFET where metal cations were applied to interact with the control layer and X_layer_ is the parameter of the EGOFET without the metal cation. The obtained data suggest that the normalized transconductance of the EGOFET sensor is associated with specific interactions between the target metal cation and the aptamer.

### 2.5. Accumulation-Based Tests for Detecting Low Concentrations of Mercury and Lead Cations

**Figure 11:**
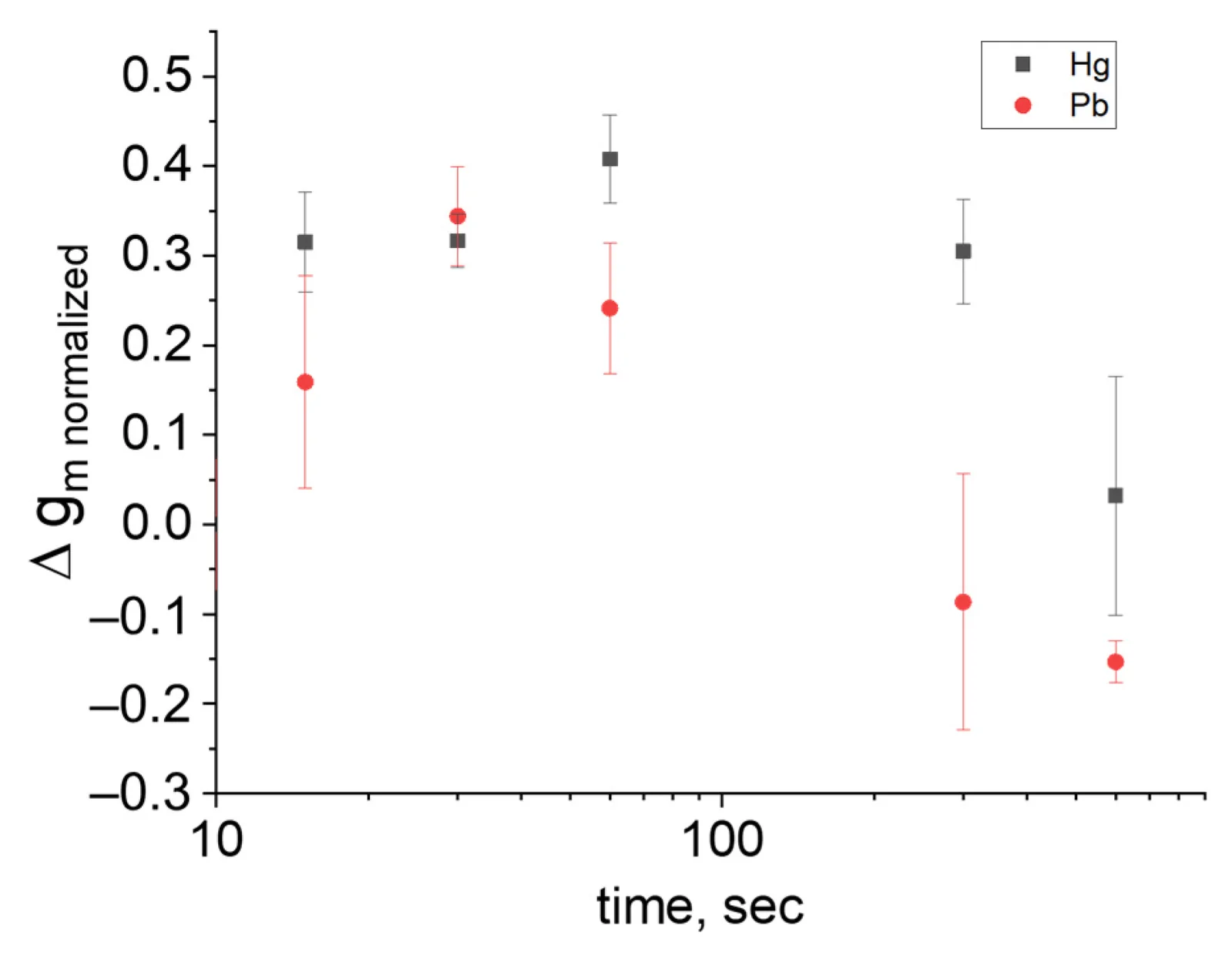
The time-dependent response of the EGOFET sensor with the HgTT aptamer to tap water containing 5 nM of Hg^2+^ or 30 nM of Pb^2+^.

The EGOFET devices were shown to be sensitive to low concentrations of lead (30 nM to 2.5 μM) and mercury (250 nM to 2.5 μM) cations. These ranges correspond to the maximum permissible concentrations for lead in fish-farming water as well as drinking and wastewater, which are 30, 144 nM and 1.2 μM, respectively. As for mercury content, the reported range is much higher than the maximum permissible concentration in drinking and wastewater, which is 5 nM. However, the EGOFET sensors are able to detect low concentrations of these cations in accumulation mode under simulated water flow conditions. To test this, we placed an EGOFET aptasensor in a beaker of tap water containing a low concentration of target or control cations under constant stirring, and measured sensor responses at 15, 30, 60, 300 and 600 s by removing the sensor from the water. Measurements were performed on the sensors with the HgTT aptamer in aqueous solutions containing 5 nM Hg^2+^ or 30 nM Pb^2+^. The beaker used had a diameter of 4 cm and a volume of 18 mL. The stirring speed was set to 1500 rpm, which corresponds to an angular velocity of 1.0 m/s, similar to the water flow velocity in a pipe, which ranges from 0.6 to 1.5 m/s. These conditions ensure that the developed sensor can accurately measure cation concentrations in a water flow. To account for device degradation and baseline drift over time, the sensor responses were calculated as the difference between responses to metal cation solutions in tap water and bare tap water: Δg_m normalized_ = g_m normalized (Me)_ − g_m normalized (tap water)_, where g_m normalized (Me)_ = g_m (Apt+Me)_/g_m (SA)_ and g_m normalized (tap water)_ = g_m (Apt+tap water)_/g_m (SA)_. The results are presented in Figure 11. The raw data of the sensor responses to target and control cations, as well as bare tap water, are shown in Figure S16 (see Supplementary Materials).

The results obtained suggest that reliable detection of 5 nM mercury is possible within 5 min after immersing the EGOFET sensor in the test solution, and it can be distinguished from the response to 30 nM lead solution. In the presence of mercury, the transconductance of the EGOFET increased compared to that observed in tap water, while in the presence of lead, it decreased compared to tap water.

## 3. Discussion

Today, traditional spectroscopic and mass spectrometry techniques are used to detect heavy metals in water (Table 4). Among the spectroscopic methods, atomic absorption spectroscopy (AAS), atomic emission spectroscopy (AES), inductively coupled plasma atomic emission spectroscopy (ICP-AES), and inductively coupled plasma mass spectrometry (ICP-MS) are the most commonly used [43]. AAS involves measuring the absorption of light after the sample has been atomized using a high-temperature flame or graphite furnace, allowing for the quantitative determination of heavy metals in water [44]. For example, the concentrations of lead, arsenic, antimony, and aluminum were measured in 50 water samples, ranging from 1 to 7.8 µg/L [45]. AAS is more effective when working with higher concentrations and larger sample volumes, but it can be slow due to the need to analyze one target at a time.

In contrast, AES is ideal for analyzing complex samples that contain multiple elements, as metal atoms are excited and emit an optical spectrum that is specific to each element. This technique uses a high-temperature atomic source, such as inductively coupled plasma (ICP), which reaches temperatures between 6000 and 10,000 K. This significantly increases the intensity of metal emissions, leading to faster analysis and higher sensitivity [46]. For example, Co^2+^, Cu^2+^, Pb^2+^, Cd^2+^, Ni^2+^, Cr^3+^, and Mn^2+^ were detected using ICP-AES in aqueous media with detection limits of 0.3–1 μg/L [47].

ICP-MS uses the ionization of metallic components in a sample to analyze the resulting charged particles based on their mass-to-charge ratio (*m*/*z*). The detection limits are about 1 ng/L–1 μg/L for heavy metals, making it suitable for the simultaneous analysis of multiple elements in complex samples with high accuracy and resolution. This is essential to the quantitative determination of heavy metals in various applications [48].

Traditional methods of heavy-metal detection provide sensitive, reliable, and reproducible measurements. These methods allow for the detection of multiple targets simultaneously, but they are limited by high cost of equipment, skilled personnel, and time required for analysis. Additionally these methods do not provide continuous analysis.

In contrast, biosensor systems provide a cost-effective, compact, and portable alternative to traditional methods. They allow for rapid analysis directly at the point of sample collection, including continuous in-flow measurements. Electrochemical biosensors are the most commonly used for heavy-metal detection. However, optical and colorimetric biosensors are also being developed. Biosensors based on aptamers are of particular interest, as these biomolecules can be easily incorporated into sensors and demonstrate high specificity in detecting heavy-metal cations. In this study, we have shown that the specificity of aptamers to Pb^2+^ and Hg^2+^ is not absolute. G-quadruplex-based aptamers to Pb^2+^ are not efficient in salt waters, due to competition with Na^+^ for the binding sites. At the same time, aptamers for Hg^2+^ have cross-specificity to Cd^2+^ ions; both cations belong to the same group in the periodic table and have similar ionic radii (1.02 Å and 0.95 Å, respectively). These off-target interactions should be considered during biosensor development and data interpretation.

The aptamer-based biosensors were successfully developed for Pb^2+^ and Hg^2+^ ion detection, with most reports using fluorescence as an analytical signal (Table 1). This study is the first to demonstrate that aptamers can be used in EGOFET sensors to specifically detect Pb^2+^ and Hg^2+^. Since the size of the analytes typical for EGOFET biosensors (proteins and viruses) significantly exceeds that of heavy-metal cations, the streptavidin concentration on the EGOFET surface was increased from 0.1 to 0.5 mg/mL to improve density of aptamer immobilization and enhance sensor sensitivity without degrading the electrical properties of the sensor. Taking into account the dimerization in some metal–aptamer complexes, the alternative method for the depositing of the metal cations onto the EGOFET surface, which involves joint incubation of cations and aptamers in solution, was also proposed. This method was shown to yield superior selectivity for mercury cations. EGOFET-based aptasensors enable the differentiation of mercury from cadmium and lead, based on divergent changes in electrical properties of EGOFETs upon interaction with target and control cations (Figure 9A,C and Figure 11). ITC measurements suggest that G-quadruplex-based aptamers cannot be used in saline waters due to competition with Na^+^ ions. Additionally, K^+^ binds to these aptamers with 4–12-fold lower affinity compared to Pb^2+^ [23,24,25]. At the same time, all accumulation experiments were conducted using tap water without any additional purification, and they demonstrated that the developed sensor is suitable for detecting target cations in tap water in the presence of other cations. The concentrations of Ca^2+^ and Mg^2+^ in tap water were 1.9–5.7 mg-ekv/L (47 nM–142 nM), while the concentrations of Fe^2+^ and Fe^3+^ were 0.1 mg/L (1.8 nM). The concentrations of Cu^2+^ and Cd^2+^ were below 1.0 mg/L and 0.001 mg/L, respectively.

**Table 4:** Comparison of biosensor configurations and limits of detection from previously reported studies. DPV is differential pulse voltammetry; EIS is electrolyte–insulator–semiconductor; CNTs is carbon nanotubes; GO is graphene oxide; SERS is surface-enhanced Raman spectroscopy; EC is electrochemical.

Target	Sensor Architecture	LoD, M	Ref.
Pb^2+^	DPV on AuNPs/thionine-modified electrode with hairpin DNA probe and Pb^2+^-induced cleavage	3.7 × 10^−10^	[49]
	Fluorescent sensor with water-soluble perylene probe and G-rich aptamer	4.8 × 10^−10^	[50]
	Label-free EIS on miniaturized Au-disk electrode with thiolated HD1 and ferricyanide probe	2.1 × 10^−8^	[51]
	DPV on Au electrode using chitosan-based molecularly imprinted polymer with embedded tetrahedral DNA nanostructure and ferricyanide probe	1.6 × 10^−8^	[52]
	Ratiometric DPV on UiO-66-CNTs/electrode using DNA aptamer complexes with G-rich aptamer and Pb^2+^-induced G-quadruplex	9.7 × 10^−9^	[53]
	Aptamer-modified EGOFET sensors	3.0 × 10^−8^	This work
Hg^2+^	Fluorescent sensor with GO quencher and FAM-labeled aptamer with partially complementary aptamer	1.1 × 10^−13^	[54]
	SERS sensor with Au substrate and dual recycling amplification with aptamer and hairpin DNA	1.1 × 10^−16^	[55]
	Dual-mode EC/fluorescence readout on 3D porous AuNPs/MXene (Ti_3_C_2_T_x_)/electrode using Nile Blue-labeled aptamer	EC: 2.7 × 10^−15^; FL: 1.6 × 10^−15^	[56]
	Label-free colorimetric sensor with aptamer-modified AgNPs	2.1 × 10^−9^	[57]
	Portable fluorescent sensor with aptamer encapsulated in PEG hydrogel disk on filter paper	4.4 × 10^−8^	[58]
	Aptamer-modified EGOFET sensors	2.5 × 10^−7^ (accumulation-based detectability is 5.0 × 10^−9^)	This work

The experiments conducted revealed that lead and cadmium can interact directly with the EGOFET surface. This was evidenced by the threshold voltage response to control cations. For the HgTT aptamer, the normalized threshold voltage parameter showed a clear dependence on Pb^2+^ concentration in the range from 100 nM to 2500 nM (Figure 9D). For the PbGQ2 aptamer, the normalized threshold voltage parameter had a dependence on Cd^2+^ concentration in the range from 100 nM to 500 nM (Figure 10B). We attribute this observation to direct interaction of Pb^2+^ and Cd^2+^ ions with the streptavidin or biotinylated receptor layers. Further investigation is needed to understand the mechanism of this interaction and the relationships between threshold voltage and physical processes on the surfaces of the device.

It should be noted that the sensors described in this study are disposable, as the recognition elements—aptamers—are located directly on the EGOFET surface and are firmly attached through biotin–streptavidin interaction. However, our previous work described reusable EGOFET-based aptasensors for influenza A virus, with an aptamer recognition layer immobilized on a disposable, easily replaceable polymer track-etched membrane positioned on the EGOFET surface [40]. Potentially, this device architecture could also be used for the detection of metal cations.

Regarding the limits of detection, they were determined using a drop of water (Figure 5A) and are not the lowest among other biosensors but are comparable to the maximum permissible concentrations of lead in fish-farming reservoirs, drinking and wastewater, which are 30, 144 nM and 1.2 μM, respectively. At the same time, the LoD for mercury was higher than the maximum permissible concentration in drinking and wastewater, which is 5 nM. To overcome this limitation, a method of accumulation-based testing for the detection of low concentrations of mercury was proposed. We placed the EGOFET sensor in a beaker containing tap water with mercury (5 nM), lead (30 nM) or cadmium (30 nM) under constant stirring and measured the sensor response at 15, 30, 60, 300 and 600 s. This system simulates testing in a water flow and enables one to obtain a sensor response by the gradual accumulation of metal cations on the EGOFET surface. This approach is possible due to the high binding constants of the selected aptamers. It was demonstrated that this approach allows us to distinguish between 5 nM mercury and 30 nM lead.

Thus, the developed biosensor’s performance is suitable for real-world applications involving the detection of mercury and lead levels exceeding the maximum permissible concentrations in drinking water and fish-farming reservoirs.

## 4. Materials and Methods

### 4.1. Materials

Synthesis of the organic semiconductor (OS) for EGOFET semiconducting-layer formation—2,7-dioctyl-benzothieno [3,2-b][1]benzothiophene (C8-BTBT-C8)—was performed as described in [59,60]. The organic semiconductor 1,3-bis{11-(7-heptyl[1]benzothieno[3,2-b][1]benzothio-2-yl)hexyl}-1,1,3,3-tetramethyl-disiloxane (BTBT-dimer) and the biotin-containing derivative of dialkyl-benzothieno[3,2-b][1]benzothiophene (BTBT-biotin) for the biotin-functionalized EGOFET bioreceptor layer were synthesized according to the routes described in [61] and [42], respectively.

Organic solvents (toluene, acetone, and isopropanol) were purchased from Acros Organics (Moscow, Russia). Polystyrene (PS) (Mw =280 000) was purchased from Sigma-Aldrich, St. Louis, MO, USA and used as received. The following reagents and solvents were used in this work: acetonitrile (GreenVan SPB LLC (Eva Science), Saint-Petersburg, Russia), pyridine (Panreac Applichem, Castellar del Valles, Spain), tetrahydrofuran (Sigma-Aldrich, St. Louis, MO, USA), dichloromethane (Pallav Chemicals & Solvents Pvt Ltd., Mumbai, India), dichloroacetic acid (Sisco Research Laboratories Pvt. Ltd., Mumbai, India), N-methylimidazole (abcr GmbH & Co KG, Karlsruhe, Germany), propionic anhydride (Sigma-Aldrich, St. Louis, MO, USA), crystalline iodine (Panreac Applichem, Castellar del Valles, Spain), aqueous ammonia solution (JSC Base No. 1 of Chemical Reagents, Moscow region, Russia), 5-ethylthio-1H-tetrazole (ChemGenes Corporation, Wilmington, MA, USA). Solvents were dried according to standard procedures. Concentrated ammonia solution was prepared by saturating an aqueous ammonia solution. Ultrapure deionized water with a resistance of 18.2 MΩ was obtained by an Akvilon deionizer D-301 (Akvilon, Moscow, Russia). Streptavidin (SA) was purchased from Sigma-Aldrich. pH values were controlled with an ST20 pH-meter (Ohaus Corporation, Parsippany, NJ, USA). Phosphate-buffered saline (PBS) consisted of 10 mM Na_2_HPO_4_, 1.8 mM KH_2_PO_4_, 137 mM NaCl, and 2.7 mM KCl, pH 7.35.

### 4.2. DNA Aptamers

The following biotinylated DNA aptamers were synthesized: (1) PbGQ, for Pb^2+^ (5′-GGG TGG GTG GGT GGG T-3′-biotin [23]); (2) PbGQ2, for Pb^2+^ (5′-GTG GGT AGG GCG GGT TGG-3′-biotin [24]); (3) HD1, for thrombin/Pb^2+^ (5′-GGT TGG TGT GGT TGG-3′-biotin [25]); (4) HgTT, for Hg^2+^ (5′-CTT TCT TCC CCG TTG TTT GTA C-3′-biotin [26]); (5) HgTT2, for Hg^2+^ (5′-TTT TTT TTT T-3′-biotin [27]). The following FAM-labeled G-quadruplexes were synthesized: PbGQl (5′-GGG (dT-6FAM-C8N3)GG GTG GGT GGG T-3′), PbGQe (5′-(FAM) GGG TGG GTG GGT GGG T-3′), HgTT6 (5′-CTT TC(dT-6FAM-C8N3) TCC CCG TTG TTT GTA C-3′), and HgTT3 (5′-CT(dT-6FAM-C8N3) TCT TCC CCG TTG TTT GTA C-3′). Also, the control oligonucleotide, Bio-RHA0385-Cy3 (biotin-5′-TT GGG GTT ATT TTG GGA GGG GGG CGG GGG TT-3′-Cy3) was used.

An unprotected glass with a controlled pore size (CPG) and a universal linker were used as a solid-phase carrier. The pore diameters were 500 and 1000 Å (AM Chemicals LLC, Vista, CA, USA). The phosphoramidites dA(N-bz) CED, dC(N-ac) CED, dG(N,N-dmf) CED and dT CED (ChemGenes Corporation, Wilmington, MA, USA) were used as monomer units. Non-nucleoside modifying reagents: 5-FAM phosphoramidite and biotin phosphoramidite (hydroxyprolinol) (Lumiprobe Corporation, Moscow, Russia). The synthesis of oligonucleotides was carried out on an automatic DNA/RNA synthesizer ASM-800ET (Biosset, Novosibirsk, Russia) according to the standard protocol of the cyanethyl phosphoramidite method.

The release of amino groups of heterocyclic bases and the phosphodiester backbone, as well as the removal of synthesized oligonucleotides from the polymer carrier, was carried out by treatment with a concentrated aqueous solution of ammonia for 15–17 h at a temperature of 55 °C. Oligonucleotides were purified using HPLC on an Agilent 1260 Infinity chromatograph with a diode array detector (DAD): a CDD-10Avp conductometric detector with a column for HPLC (Sol EC Nucleosil 300-5 C4 250 mm × 4.6 mm, 5 microns, 300 Å), manufactured by Macherey-Nagel, Düren, Germany (Sorbent—C4; particle size of sorbent is 5 microns, the pore size of sorbent particles is 300 Å, the inner diameter of the column is 4.6 mm, and the column length is 250 mm). Mobile phase A contained 0.2 M triethylamine in 0.2 M acetic acid, pH 6.5; mobile phase B was prepared by mixing acetonitrile with mobile phase A (80:20 *v*/*v*). Purification was performed at a rate of 1 mL/min at room temperature with detection by absorbance at 260 nm. The purified aptamers were lyophilized and stored at −20 °C.

For the proper folding of their tertiary structure, aptamers were folded at 2–100 μM concentration in Tris-HNO_3_-based buffers. The concrete buffers are described in the following subsections. The folding process was carried out in the following way: the solution was heated to 95 °C for 5 min and allowed cooled at room temperature.

### 4.3. Isothermal Titration Calorimetry

As reaction buffers, we used 20 mM Tris-HNO_3_ (pH 7.0) or 20 mM Tris-HNO_3_ + 140 mM NaNO_3_ (pH 7.0). Aptamer solutions (3 μM) and 100 μM solutions of target metal salts (Pb(NO_3_)_2_, HgCl_2_) and a control metal salt (Cd(NO_3_)_2_) were prepared. The thermal effect of aptamer binding to metal cations was measured at 25 °C using a VP-iTC calorimeter (MicroCal Ltd., Piscataway, NJ, USA). Titration was performed using sequential 10 μL injections with 5 min interval between injections. The measured heat values were corrected for ligand dilution effects. Data processing was performed in Origin 7.0 (OriginLab, Northampton, MA, USA) using the MicroCal ITC subroutine for single- and dual-binding site models.

### 4.4. Circular Dichroism

Aptamers at a concentration of 1–2 μM in 20 mM Tris-HNO_3_ buffer, pH 7.0, were placed in a quartz cuvette with a 1 cm optical path. Circular dichroism spectra were recorded using a Chirascan circular dichroism spectrometer (Applied Photophysics, Leatherhead, UK) and an MOS-500 spectrometer (BioLogic, Seyssinet-Pariset, France) equipped with a thermoelectric temperature controller at 20 °C. The wavelength range was within 220–360 nm. The buffer spectrum was subtracted as a baseline.

Aptamer denaturation experiments were performed in the temperature range of 10–90 °C at an average heating rate of 0.5 °C/min. The melting temperature was determined by fitting the obtained dependence using the Boltzmann function in Origin 8.0 software (OriginLab, Northampton, MA, USA).

### 4.5. Stopped-Flow Kinetic Measurements

Stopped-flow kinetic experiments were performed using an MOS-200 Fast Kinetics Optical System equipped with an SFM-3000/S stopped-flow mixer (BioLogic Science Instruments, Seyssinet-Paris, France). Aptamer solution at 2 or 4 μM was placed in one syringe, and a 2 μM solution of the corresponding target cation or a 1 mM solution of a non-specific metal ion was placed in the other. The solutions were mixed in different stoichiometries. Estimated dead times were in the range of 1.1–1.9 ms. The excitation wavelength was 494 nm, the emission spectrum was cut off with a 530 nm filter. Fluorescence was averaged for 1 ms (1 point) during 10–20 s and for 0.2 ms (1 point) during 4 s.

### 4.6. Fabrication of EGOFET Devices

Heavily doped n-type silicon wafers with thermally grown SiOx (300 nm thick), pre-cleaned in solvents were used as substrates for device fabrication. Gold interdigitated source and drain electrodes (channel length L = 50 µm, channel width W = 18,230 µm; W/L ratio = 364.6) were deposited via thermal evaporation through a shadow mask. Subsequently, the wafers with the electrodes were treated with oxygen plasma for 5 s and then surface-modified by immersing the substrate for 15 min in a 15 mM 2,3,4,5,6-pentafluorothiophenol solution in isopropanol following the procedure in [62]. Deionized water (18.2 MΩ) served as the electrolyte. A platinum wire immersed in the electrolyte functioned as the gate electrode. A C8-BTBT-C8:PS mixture (4:1 weight ratio) was prepared by dissolving C8-BTBT-C8 in a toluene solution of polystyrene in order to obtain a total concentration of 18 mg/mL. Blend deposition was made with Doctor Blade coater Film Applicator Coatmaster 510 (Erichsen, Hemer, Germany) at 85 °C as described in [63] and resulted in a crystalline film of the organic semiconducting layer. Solutions of BTBT-dimer (C = 0.33 mg/mL, in toluene) and BTBT-biotin (C = 0.33 mg/mL, in toluene) were mixed in a weight ratio of 7:3 and then used to fabricate the biorecognition layer containing of 30% BTBT-biotin on the OS layer surface. The Langmuir–Schaeffer technique of biorecognition-layer fabrication was described earlier in [43].

The storage stability of the EGOFET devices was addressed in our previous work [64]. EGOFET-type devices incorporating C8-BTBT-C8 and D2-Und-BTBT-Hex semiconductor layers retain high-performance characteristics after being stored under laboratory conditions for 3 weeks and 4 months, respectively; such devices can be used for the application of a recognition layer and subsequently as biosensors, even after two months of storage under laboratory conditions.

### 4.7. EGOFET Surface Characterization

The formation of the semiconducting and biorecognition layers and their further modification were controlled by an NT-MDT Solver Next atomic force microscope (NT-MDT, Moscow, Russia) in tapping mode under ambient conditions using NT-MDT HA-FM silicon probes with a resonant frequency of 77 kHz. Polarizing optical microscopy was conducted at all stages of the experiments using Carl Zeiss Axioscop A40Pol (Carl Zeiss, Oberkochen, Germany).

### 4.8. Electrical Measurements

The electrical measurements were performed using a Keithley 2634B (Keithley, Beaverton, OR, USA) source meter in an ambient environment using a probe station PS-100 (Printeltech LLC, Moscow, Russia). A platinum wire (Ø 0.5 mm) was used as a gate electrode during measurements in the drop on the EGOFET surface. A delay of 100 ms between the drain/gate voltage sourcing and current measurement was set to avoid capacitive effects. Every curve was measured in direct and reverse scans, which consisted of 41 points in each direction. Field-effect mobility values were extracted by fitting the transfer characteristics with a Shockley’s gradual-channel model in saturation mode (V = −0.5 V) (1):

(1) $$I_{\mathrm{DS}}=\mu \frac{WC_{i}}{2L}\left.{\left(V_{\mathrm{GS}}-V_{\mathrm{th}}\right)}^{2}\right.$$

where I_DS_ is the source-drain current; µ is the charge carrier mobility; W and L are the channel width and length, respectively; C_i_ is the capacitance per unit area, Vth is the threshold voltage, V_GS_ is the gate-source voltage and V_DS_ is the drain-source voltage [61]. The transconductance in the saturated regime was calculated as follows [62]:

(2) $$g_{m}=\frac{{\partial \surd I}_{\mathrm{DS}}}{{\partial V}_{\mathrm{GS}}}$$

The number of replicate measurements used for error bars for each concentration of each cation was not less than four different EGOFET devices.

### 4.9. EGOFET Aptasensor Assembly and Performance

To establish the peak performance conditions for the sensor, the following oligonucleotide concentrations were studied directly on the EGOFET surface: 2 µM, 20 µM and 100 µM. The Bio-RHA0385-Cy3 oligonucleotide featuring a 5′-biotin modification for immobilization and a 3′-Cy3 fluorescent tag for detection was employed to evaluate the bioreceptor layer’s attachment to the EGOFET substrate. Fluorescence analysis was conducted using a FotonBio Raman spectrometer (FotonBio, Moscow, Russia) equipped with a 532 nm laser, capturing spectra between 230 and 4000 cm^−1^.

Streptavidin and oligonucleotide stocks were prepared in PBS (pH 7.3). Oligonucleotide solutions (20 and 2 μM) underwent a thermal denaturation process (95 °C for 5 min) followed by cooling to room temperature, with subsequent dilutions performed in DMSO. A calibration curve was established using aptamer concentrations from 0 to 20 nM in DMSO. The theoretical maximum for a completely filled monolayer was calculated according to the suggestion that the area of the aptamer’s layer on the surface equals to the area of the drop, which was 7.1 mm^2^. The fraction of occupied adsorption sites for a theoretical monolayer was calculated, giving the aptamer diameter of 3 nm (this value is explained in Figure S14; see Supplementary Materials) [65]; in this case, an ideal monolayer of aptamer will contain 0.235 pmoles per mm^2^ of EGOFET surface.

**Figure 12:**
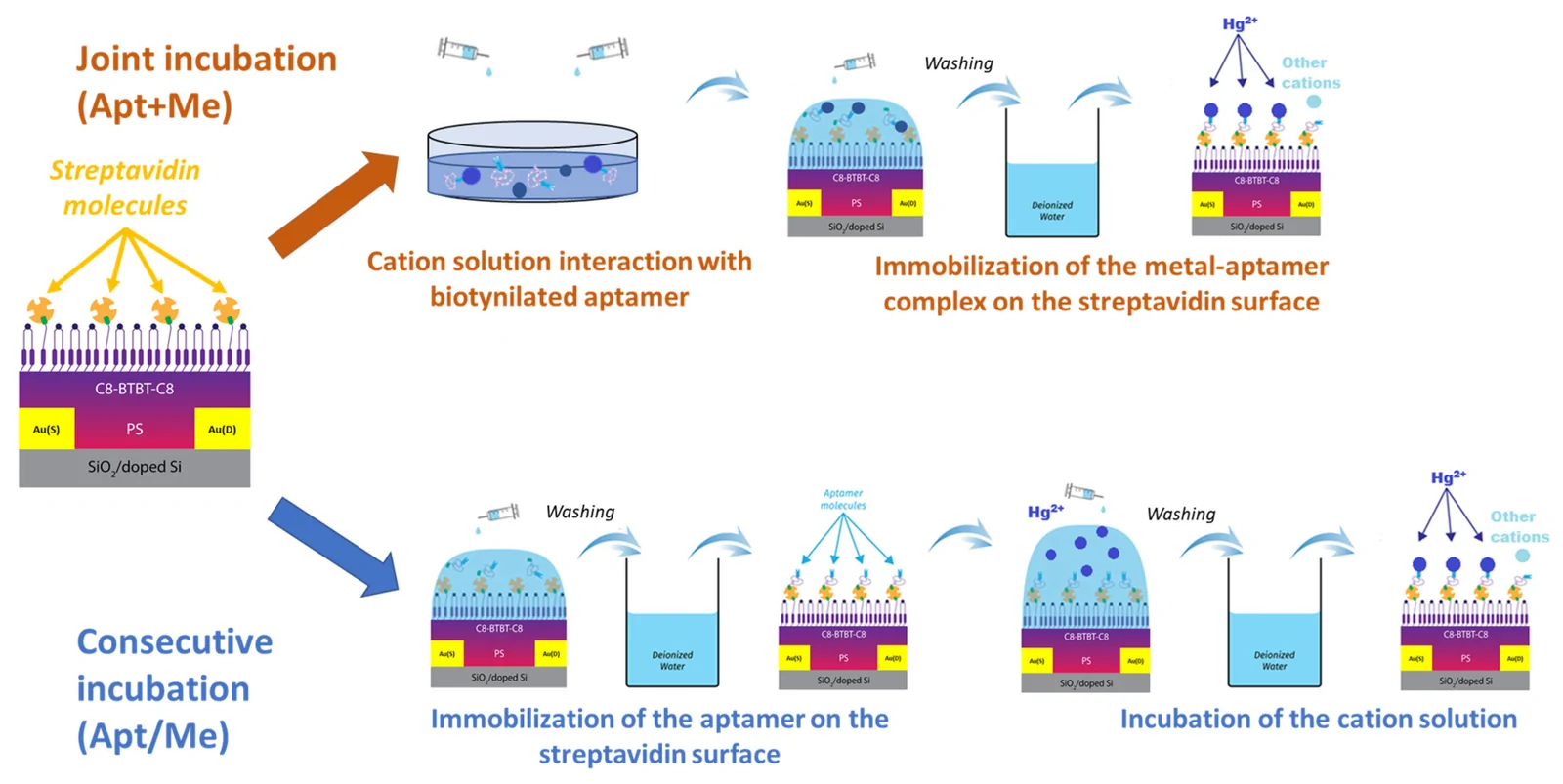
Scheme of the possible immobilization techniques for heavy-metal cation detection—joint and consecutive incubation of metal cations and recognition elements.

For the binding analysis, 5 μL of streptavidin solution (500 mg/L) was incubated on the substrate for 20 min, followed by rinsing with buffer and water. Subsequently, 5 μL of oligonucleotide solution (0.2, 2, or 20 μM) was applied, incubated for 10 min, and then washed away. The bioreceptor layer was then removed by immersing the substrate in DMSO and centrifuging for 5 min to ensure complete dissolution. The fluorescence of the supernatant was then measured. To account for substrate-induced signal interference, control measurements were performed by adding oligonucleotides (0.01–20 nM) to a DMSO solution containing the material washed off from a streptavidin-coated substrate. To prepare the biorecognition layers, EGOFETs were processed under optimized conditions according to the following protocol: 1) immobilization of streptavidin (500 mg/L solution in PBS, pH = 7.4), followed by rinsing with PBS and water after a 20-min incubation period; 2) immobilization of biotinylated aptamers (2 µM solution in 20 mM Tris-HNO_3_, pH = 7.0), followed by rinsing with 20 mM Tris-HNO3 buffer and water after a 10-min incubation period. Subsequently, aqueous solutions of metal cations were applied to the EGOFET surface for 3 min, and then the surface was rinsed with water—this immobilization technique was called consecutive. This approach limits the degrees of freedom of aptamer molecules on the EGOFET surface and affects the efficiency of metal binding and aptamer–metal complex formation—particularly in the case of non-stoichiometric complexes. Alternatively, a “co-immobilization” method was employed: the aptamer–cation complex was assembled in solution and then immobilized onto the streptavidin layer instead of the initial aptamer. This immobilization technique was called joint and allowed the aptamer to interact freely with the metal in solution, with the resulting complex being subsequently anchored to the EGOFET surface. The schemes of both techniques are presented in Figure 12. The procedures provide the specific binding of every layer.

The performance of EGOFET-based sensors was evaluated based on their ability to distinguish between target (Pb^2+^ and Hg^2+^) and control (Cd^2+^) cations. This study was conducted over concentration ranges of 100 nM to 2.5 μM (for Pb^2+^) and 100 nM to 4 μM (for Hg^2+^). To test the capability of the EGOFET sensors to detect low concentrations of mercury and lead, experiments based on an accumulation method were performed. EGOFET sensors with the corresponding aptamer layer applied to their surface were placed in a beaker containing tap water spiked with 30 nM Pb^2+^ or 5 nM Hg^2+^ as well as 30 nM Cd^2+^. The sensors were left in the solution for 15, 30, 60, 300 and 600 s and then removed from the beaker, and their electrical properties were measured.

## 5. Conclusions

Summarizing the data obtained, we have demonstrated for the first time that aptamers can be used in EGOFET sensors to detect Pb^2+^ and Hg^2+^ in liquid media in a simple, fast, specific and low-cost manner. Despite the fact that EGOFET-based aptasensors have traditionally been used to detect larger biomolecules such as viruses and proteins, they also hold promise for solving the problem of detecting heavy metals. To realize this potential, we chose several aptamers with high affinity for and specificity to Pb^2+^ and Hg^2+^. We determined the dissociation constants and stoichiometry of their complexes with target and control cations, investigated the kinetics of complex formation, and determined cross-specificity. We also proposed a putative structure for the aptamer–cation complexes. Based on these aptamers we constructed EGOFET-based aptasensors for Pb^2+^ and Hg^2+^. Since the size of analytes typical for EGOFET biosensors, such as proteins and viruses, significantly exceeds that of heavy-metal cations, and considering the dimerization in several metal–aptamer complexes, we increased the streptavidin concentration on the EGOFET surface and optimized both the aptamer concentration and the method of analyte capture by the EGOFET surface to increase sensor sensitivity. The fabricated EGOFET devices were shown to be able to detect small concentrations of lead (from 30 nM to 2.5 μM) and mercury (from 250 nM to 2.5 μM) cations. These correspond to the maximum permissible concentrations of lead in drinking and wastewater of 144 nM and 1.2 μM, respectively, but are higher than the maximum permissible concentration in drinking and wastewater for mercury which is 5 nM. Despite this, the possibility of detecting mercury cations at a concentration of 5 nM in 5 min in a model system simulating liquid flow was demonstrated using the fabricated EGOFET. The limits of detection for standard measurements in the droplet were determined as 30 nM for lead cations and 250 nM for mercury cations. These values are essentially limited by the amount of target cations present within the 2.5 µL droplet of analyzed solution. At the same time, in accumulation mode, the sensor response is determined by the gradual accumulation of metal cations bound to the aptamers on the EGOFET surface due to the high binding constants of the chosen aptamers and is limited only by the rate of device degradation in water flow. Therefore, it allows for the accumulation-based detection of 5 nM mercury cations to be achieved. It should also be noted that the analysis takes no more than 10 min, does not require complex sample preparation (one can use a drop of tap or drinking water without any labeling), and can be performed outside the laboratory using the portable device described in [39]. The final device’s low cost is based on commercially available, low-cost C8-BTBT organic semiconductor and aptamers, as well as a very small amount of material (only a few nanograms) required to produce one sensor. Silicon substrates can be replaced with cheap glass wafers. EGOFET sensors are designed to be compatible with scalable, low-cost solution processing and printing fabrication techniques (such as inkjet printing, spin coating or dr. Blade, as applied in this study), which makes the fabrication cost approximately one dollar per device for high-volume production. So, the proof of concept for fast and selective detection of Pb^2+^ and Hg^2+^ ions at maximum permissible concentrations in drinking water and fish-farming reservoirs using an EGOFET-based aptasensor has been implemented for the first time, paving the way for flow sensors for heavy-metal detection in liquid media.

## Figures and Tables

**Table 1:** Dissociation constants and stoichiometries of aptamer–metal complexes with target and off-target cations, as determined by ITC.

	PbGQ	PbGQ2	HD1	HgTT		HgTT2
	Pb^2+^ (target cation)			Hg^2+^ (target cation)		
K_D_, nM	270 ± 11	120 ± 4	19 ± 11	1st step 350 ± 40	2nd step 760 ± 8	620 ± 40
Stoichiometry aptamer:metal	2:1	1:2	1:1	2:1	2:6	2:5
	Cd^2+^ (off-target cation)					
K_D_, nM		10,000 ± 3000				1160 ± 100

**Table 2:** Dissociation constants (K_D_) of complexes formed between FAM-labeled aptamers and different cations.

K_D_, nM (Stoichiometry Aptamer:Metal)				
	PbGQl	PbGQe	HgTT3	HgTT6
Pb^2+^	34 ± 2 (1:1)	33 ± 1 (2:1)	29,000 ± 500 (2:1)	350,000 ± 40,000 (2:1)
Hg^2+^	330,000 ± 8000 (2:1)	28,000 ± 10,000 (1:2)	1360 ± 200 (2:1)	300 ± 50 (2:1)
Cd^2+^	31,000 ± 4000 (2:1)		1130 ± 30 (2:1)	910,000 ± 180,000 (2:1)
Ni^2+^	360,000 ± 70,000 (2:1)	660,000 ± 19,000 (2:1)		
Sr^2+^	250,000 ± 12,000 (2:1)	404,000 ± 15,000 (1:2)		
Co^2+^	100,000 ± 30,000 (2:1)			136,000 ± 13,000 (1:3)

**Table 3:** Saturation of the EGOFET surface with fluorescently labeled oligonucleotides, depending on their concentration during immobilization.

Oligonucleotide Concentration During Immobilization, μM	Oligonucleotide Concentration in DMSO Flushes, nM	The Fraction of Occupied Adsorption Sites for a Theoretical Monolayer, %
0.2	0.0468 ± 0.0005	1.41
2	0.211 ± 0.008	6.37
20	0.67 ± 0.05	20

## Data Availability

The completed data of this study are available from the corresponding author.

## Supplementary Materials

The following supporting information can be downloaded at https://www.mdpi.com/article/10.3390/ijms27219711/s1.

## Abbreviations

The following abbreviations are used in this manuscript:

EGOFET	Electrolyte-gated field-effect transistor
C_8_-BTBT-C_8_	2,7-dioctyl-benzothieno[3,2-b][1]benzothiophene
BTBT-biotin	biotin-containing derivative of dialkyl-benzothieno[3,2-b][1]benzothiophene
BTBT-dimer	1,3-bis{11-(7-heptyl[1]benzothieno[3,2-b][1]benzothio-2-yl)hexyl}-1,1,3,3-tetramethyl-disiloxane
