# Supplementary material for: Simple, Fast and Cheap Pb2+ and Hg2+ Detection in Liquid Media Using EGOFET-Based Aptasensors
Source: Int J Mol Sci. 2026 Oct 29;27(21):9711. doi: 10.3390/ijms27219711 (PMC13763115; doi:10.3390/ijms27219711)
Supplement: Supplementary file 1 [file ijms-27-09711-s001.zip › ijms-4607183-supplementary.pdf]

Supplementary Materials

Simple, fast and cheap Pb^{2+} and Hg^{2+} detection in liquid media with EGOFET-based aptasensors

Anna K. Keshek[1,2], Dmitriy A. Skvortsov[1,3], Elena Yu. Poimanova[1,3], Victor N. Orlov[4], Ilya P. Oleynikov[4], Margarita S. Stiazhkina[5], Askar D. Turashev[5], Elena G. Zavyalova[1,2], Elena V. Agina[1,3,*]

[1]Enikolopov Institute of Synthetic Polymeric Materials of Russian Academy of Sciences, Profsoyuznaya Str. 70, 117393 Moscow, Russian Federation; dmitriiskv141@gmail.com (D.A.S.); poymanova@ispm.ru (E.Y.P.)
[2]Lomonosov Moscow State University, Chemistry Department, Leninskiye gory 1/3, 119991 Moscow, Russian Federation; akeshek@list.ru (A.K.K.); zlenka2006@gmail.com (E.G.Z.)
[3]Lomonosov Moscow State University, Department of Fundamental Physical and Chemical Engineering, Leninskiye Gory 1/51, 119991 Moscow, Russia; werdas@mail.ru (E.V.A.)
[4]Lomonosov Moscow State University, Belozersky Institute of Physico-Chemical Biology, Leninskiye gory 1/40, 119991 Moscow, Russian Federation; orlovvn@belozersky.msu.ru (V.N.O.); oleynikov.biophys@gmail.com (I.P.O.)
[5]Central Research Institute of Epidemiology, Federal Service for Surveillance on Consumer Rights Protection and Human Wellbeing Russian Federation, Novogireevskaya Str. 3a, 111123 Moscow, Russia; stiazhkina@cmd.su (M.S.S.); turashev@cmd.su (A.D.T.)
*Correspondence: werdas@mail.ru

Content:

Figure S1. ITC spectra of 3 μM aptamer + 100 μM metal solution in 20 mM Tris-HNO_3 + 140 mM $NaNO_3$ buffer, pH 7.0, 25°C: (A) PbGQ + $Pb(NO_3)_2$, (B) PbGQ2 + $Pb(NO_3)_2$, (C) HgTT2 + $HgCl_2$. 3

Figure S2. ITC spectra of 3 μM aptamer + 100 μM $Cd(NO_3)_2$ in 20 mM Tris-HNO_3 buffer, pH 7.0, 25°C: (A) PbGQ2, (B) HgTT2..........3

Figure *S*3. CD spectra for 1 μM aptamer PbGQ: (A) denaturation without Pb^{2+} cations; (B) denaturation in 10 μM Pb^{2+} cations. 4

Figure *S*4. CD spectra for 1 μM aptamer PbGQ2: (A) denaturation without Pb^{2+} cations; (B) denaturation in 100 μM Pb^{2+} cations..........4

Figure S5. CD spectra for 2 μM aptamer HD1: (A) denaturation without Pb^{2+} cations; (B) denaturation in 100 μM Pb^{2+} cations. 4

Figure *S*6. CD spectra for aptamer 1 μM HgTT: (A) denaturation without Hg^{2+} cations; (B) denaturation in 50 μM Hg^{2+} cations. 5

Figure *S*7. CD spectra for 2 μM aptamer HgTT2: (A) denaturation without Hg^{2+} cations; (B) denaturation in 50 μM of Hg^{2+} cations. 5

Figure *S*8. CD spectra for 2 μM aptamer PbGQl: (A) denaturation without Pb^{2+} cations; (B) denaturation in 50 μM Pb^{2+} cations..........5

Figure *S*9. CD spectra for 2 μM aptamer PbGQe: (A) denaturation without Pb^{2+} cations; (B) denaturation in 50 μM Pb^{2+} cations..........6

Figure *S*10. CD spectra for 2 μM aptamer with Hg^{2+} cations concentrations in the range 0-16 μM: (A) HgTT3; (B) HgTT6..........6

Figure S11. Transfer characteristics of the EGOFET device with streptavidin layer, shown for 20 devices on the substrate..........6

Figure S12. Fluorescence and Raman spectra of the fluorescent oligonucleotide at different concentrations: (A) oligonucleotide in DMSO; (B) oligonucleotide with a DMSO wash from EGOFET.......7

Figure S13. Morphology of the EGOFET surfaces: POM-images of a single EGOFET pixel with organic semiconducting (A) and bioreceptor (B) layers, AFM-images of the EGOFET biotinylated surface (C) and the EGOFETs with streptavidin (D) and aptamer PbGQ (E) and HD1 (F) top layers: aptamers. 8

Figure S14. Estimation of DNA aptamer size. The width of three-quartet G-quadruplex aptamer (pdb id 6PNK [1]) was 26.3 and 32.5 Å as estimated using PyMol software. 9

Figure S15. Dependence of the EGOFET sensor response on the target Hg^{2+} (A) and Pb^{2+} (B, C) cations concentration for $g_{m\ normalized}$ (A, B) and $V_{th\ normalized}$ (C) measured for the EGOFET modified with the aptamer HgTT specific to Hg^{2+} (A) the aptamer PbGQ2 specific to Pb^{2+} (B, C) with a confidence interval of 0.95..10

Table S1. Regression equations parameters, including intercepts and calibration slopes of the calibration curves for Hg^{2+} and Pb^{2+} with corresponding aptamers.................................... **Error! Bookmark not defined.**

Figure S16. Time-dependent response of the EGOFET sensor based on the PbGQ2 aptamer to (A) Pb^{2+} (C=30 nM), (B) Cd^{2+} (C=30 nM) solutions in tap water, (C) to bare tap water..11

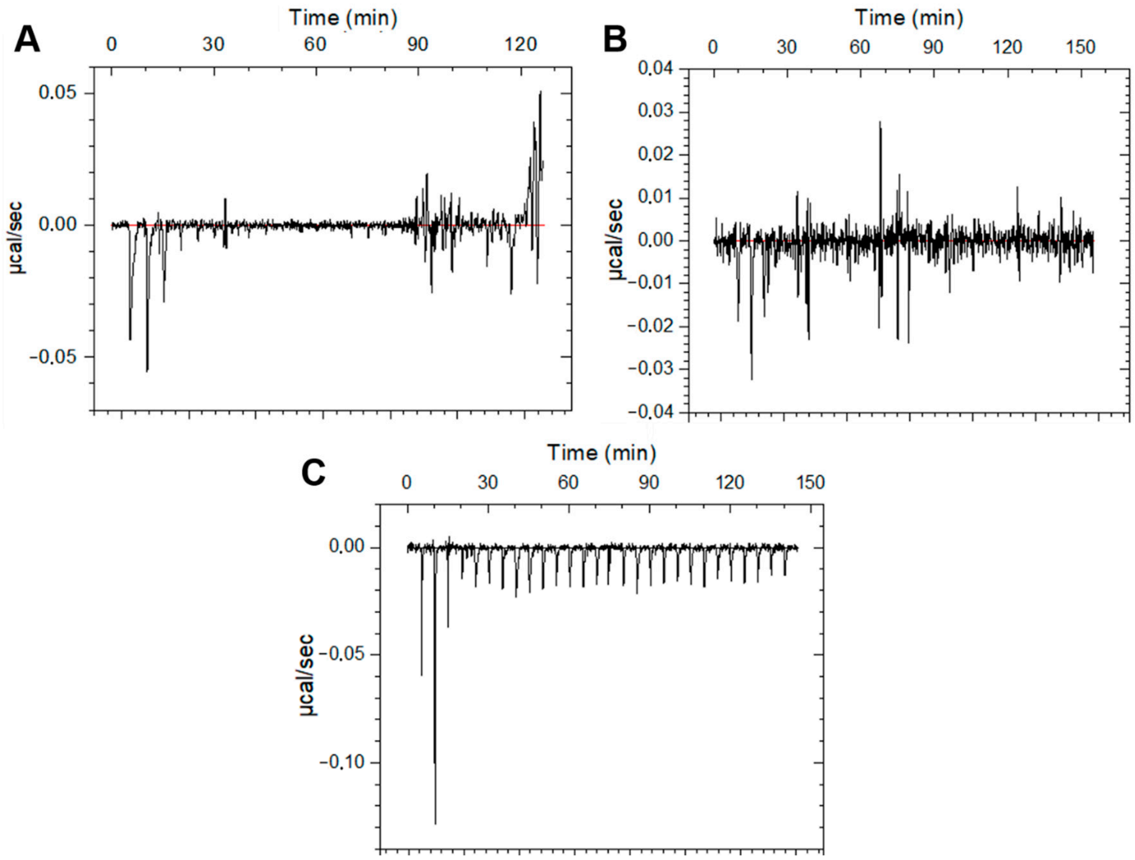

Figure S1. ITC spectra of 3 µM aptamer + 100 µM metal solution in 20 mM Tris-HNO_3 + 140 mM $NaNO_3$ buffer, pH 7.0, 25°C: (A) PbGQ + $Pb(NO_3)_2$, (B) PbGQ2 + $Pb(NO_3)_2$, (C) HgTT2 + $HgCl_2$.

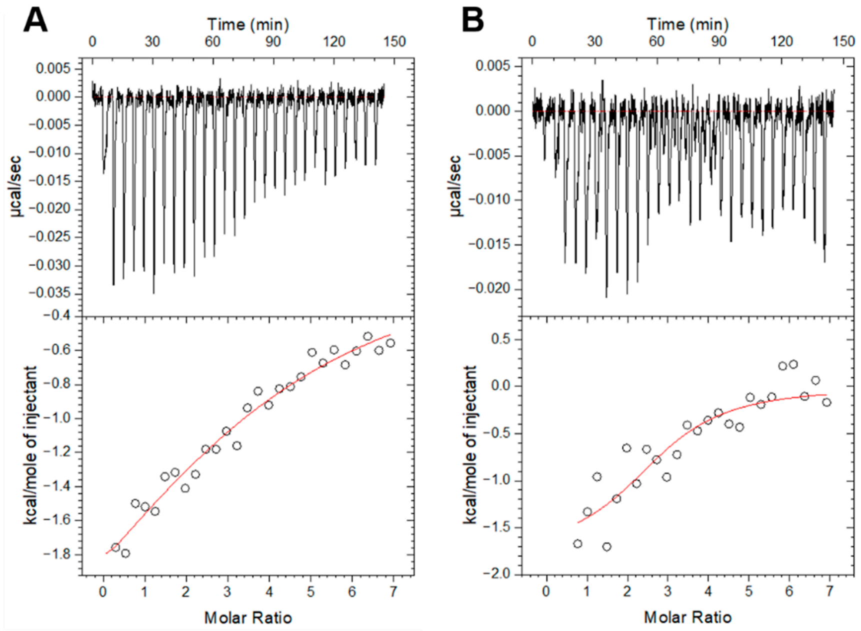

Figure S2. ITC spectra of 3 µM aptamer + 100 µM $Cd(NO_3)_2$ in 20 mM Tris-HNO_3 buffer, pH 7.0, 25°C: (A) PbGQ2, (B) HgTT2.

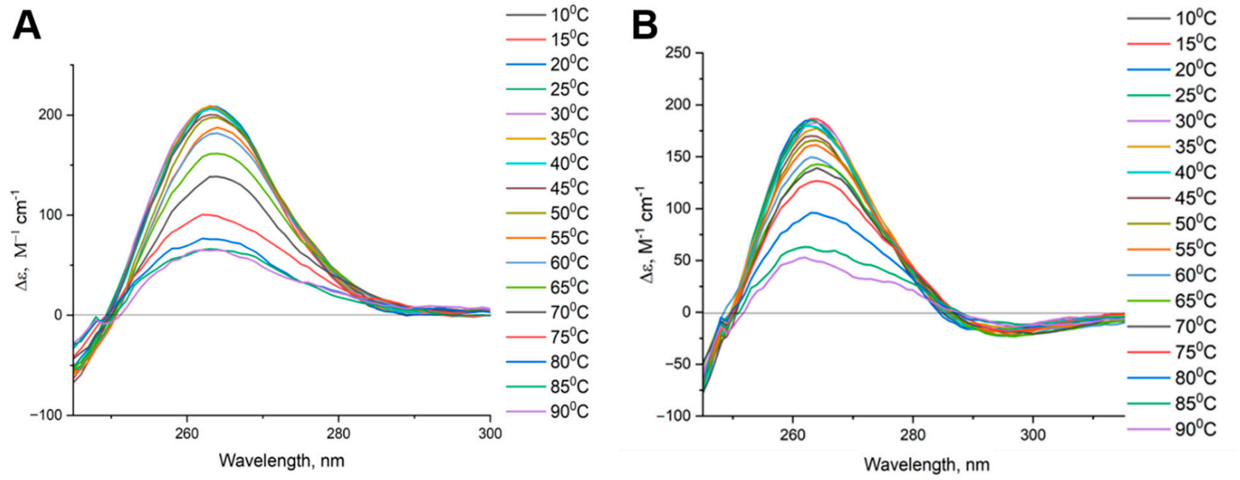

Figure *S*3. CD spectra for 1 μM aptamer PbGQ: (A) denaturation without Pb^{2+} cations; (B) denaturation in 10 μM Pb^{2+} cations.

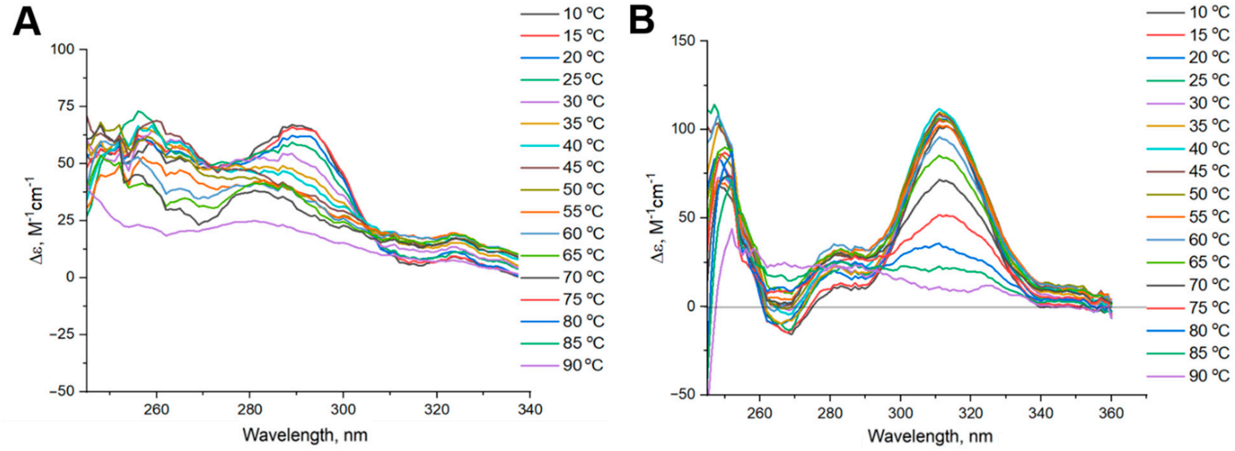

Figure *S*4. CD spectra for 1 μM aptamer PbGQ2: (A) denaturation without Pb^{2+} cations; (B) denaturation in 100 μM Pb^{2+} cations.

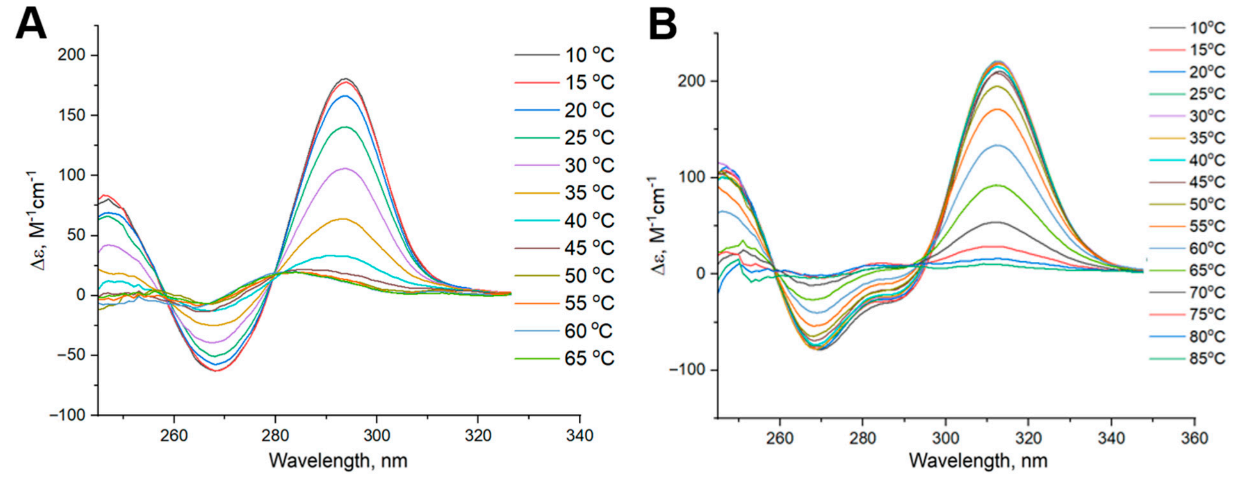

Figure S5. CD spectra for 2 μM aptamer HD1: (A) denaturation without Pb^{2+} cations; (B) denaturation in 100 μM Pb^{2+} cations.

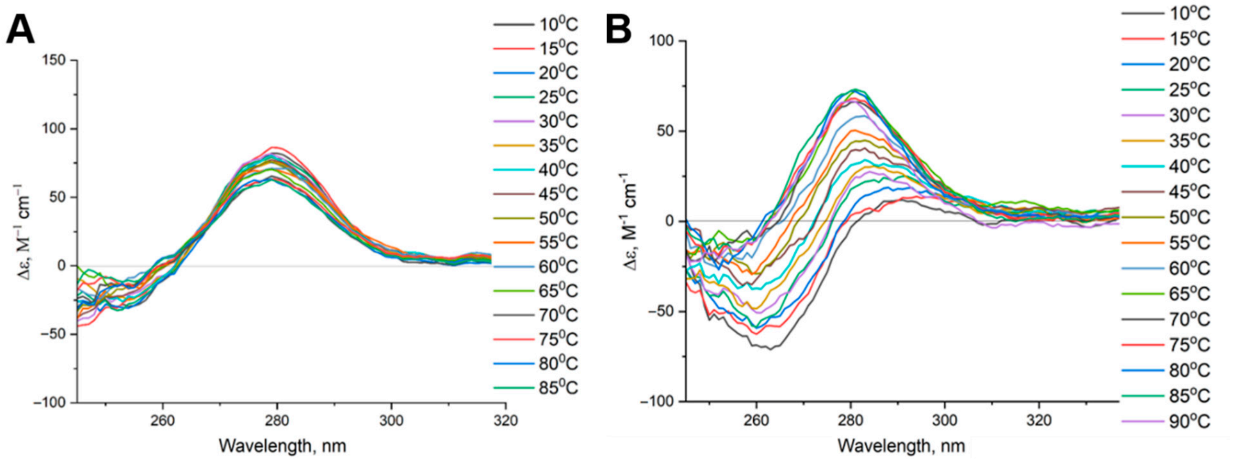

Figure S6. CD spectra for aptamer 1 μM HgTT: (A) denaturation without Hg^{2+} cations; (B) denaturation in 50 μM Hg^{2+} cations.

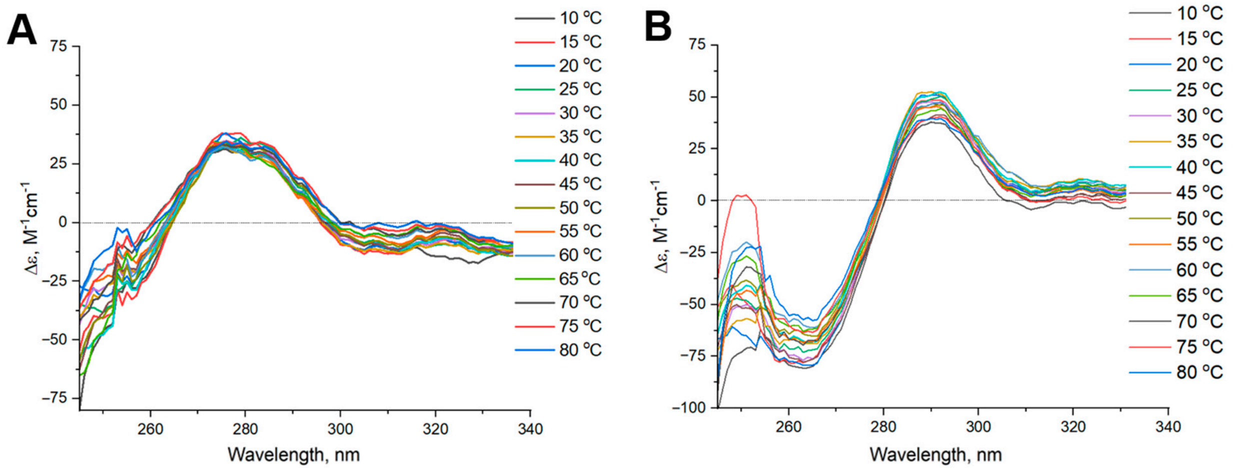

Figure S7. CD spectra for 2 μM aptamer HgTT2: (A) denaturation without Hg^{2+} cations; (B) denaturation in 50 μM of Hg^{2+} cations.

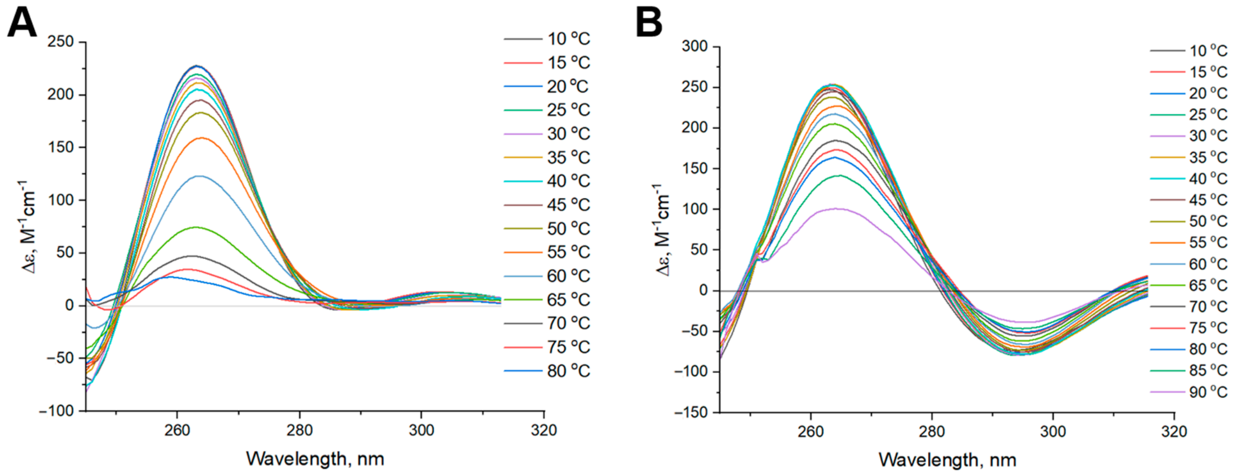

Figure S8. CD spectra for 2 μM aptamer PbGQl: (A) denaturation without Pb^{2+} cations; (B) denaturation in 50 μM Pb^{2+} cations.

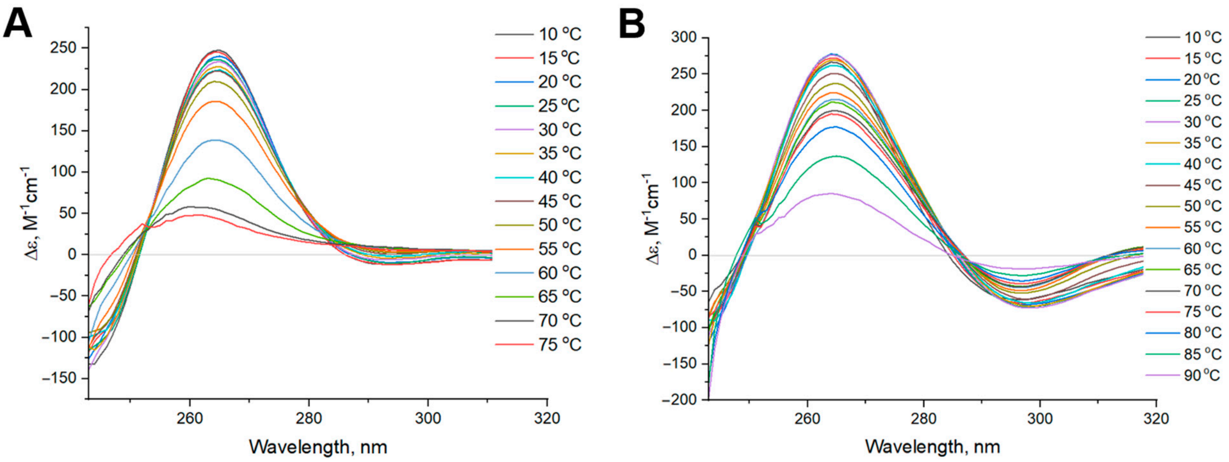

Figure *S*9. CD spectra for 2 μM aptamer PbGQe: (A) denaturation without Pb^{2+} cations; (B) denaturation in 50 μM Pb^{2+} cations.

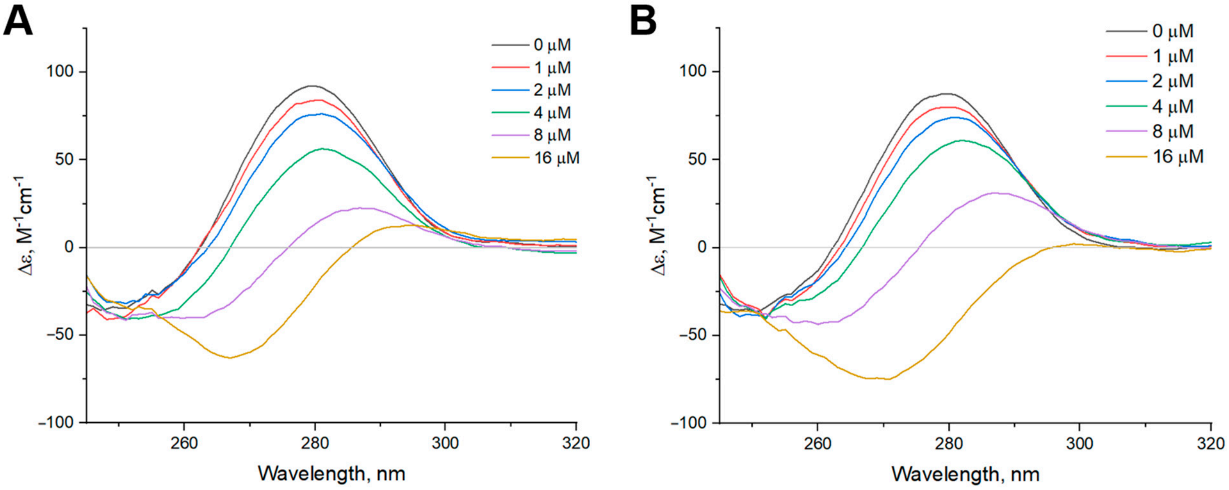

Figure *S*10. CD spectra for 2 μM aptamer with Hg^{2+} cations concentrations in the range 0-16 μM: (A) HgTT3; (B) HgTT6.

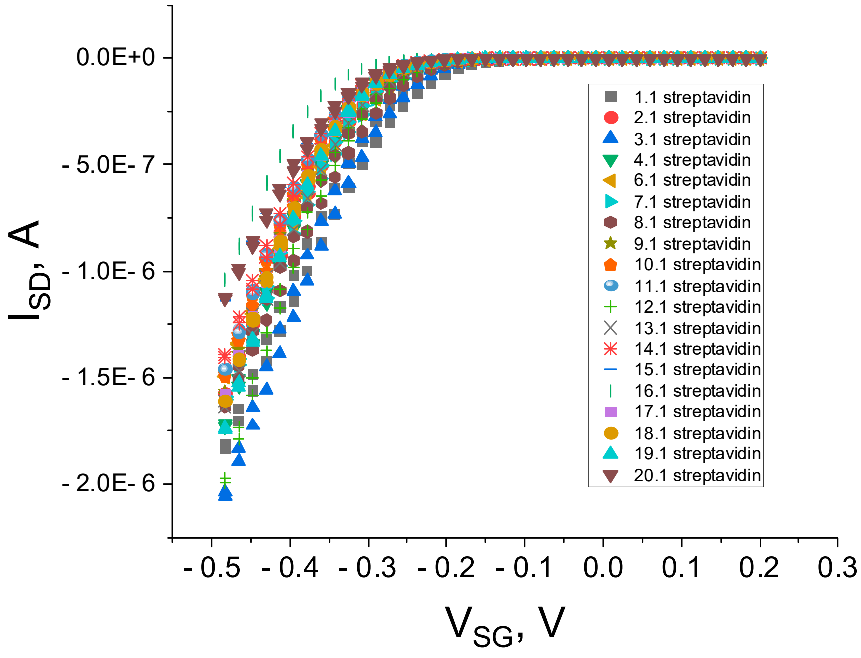

Figure S11. Transfer characteristics of the EGOFET device with streptavidin layer, shown for 20 devices on the substrate.

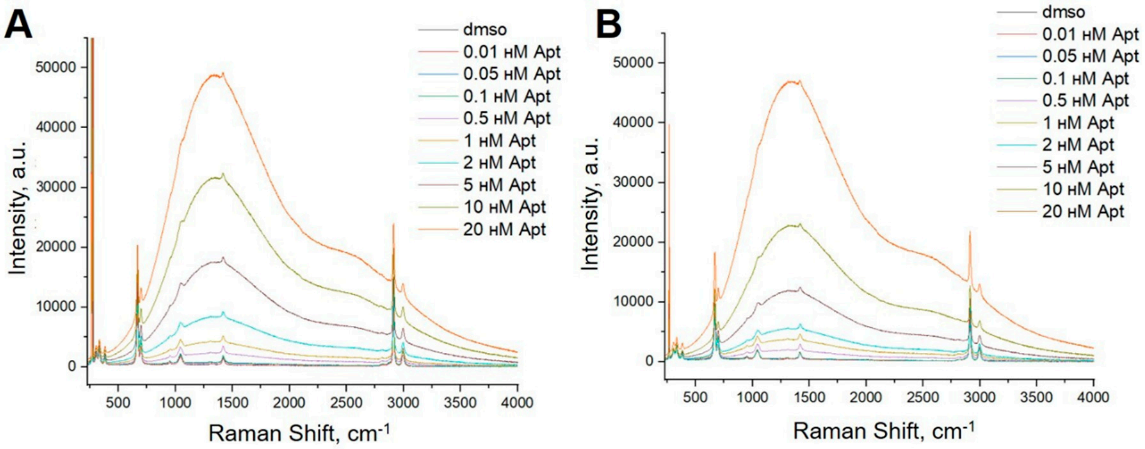

Figure S12. Fluorescence and Raman spectra of the fluorescent oligonucleotide at different concentrations: (A) oligonucleotide in DMSO; (B) oligonucleotide with a DMSO wash from EGOFET.

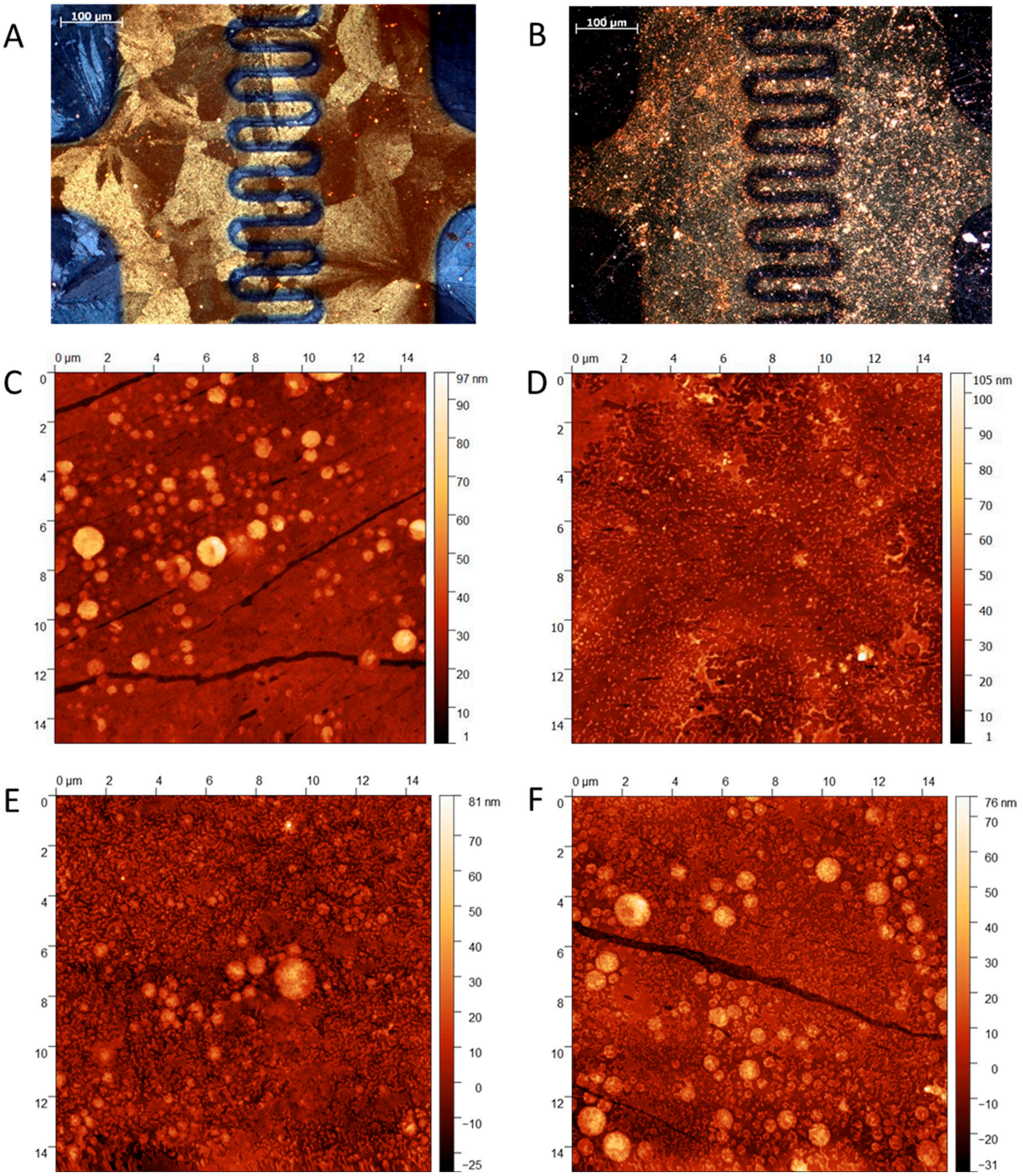

Figure S13. Morphology of the EGOFET surfaces: POM-images of a single EGOFET pixel with organic semiconducting (A) and bioreceptor (B) layers, AFM-images of the EGOFET biotinylated surface (C) and the EGOFETs with streptavidin (D) and aptamer PbGQ (E) and HD1 (F) top layers: aptamers.

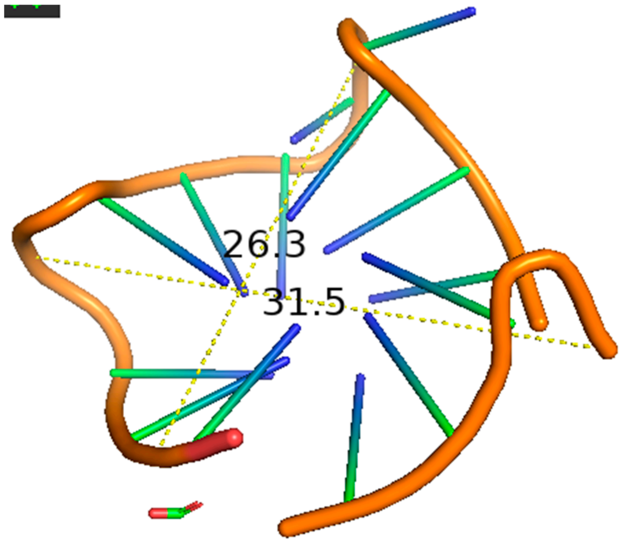

Figure S14. Estimation of DNA aptamer size. The width of three-quartet G-quadruplex aptamer (pdb id 6PNK [1]) was 26.3 and 31.5 Å as estimated using PyMol software.

References

1. Lin L.Y., McCarthy S., Powell B.M., Manurung Y., Xiang I.M., Dean W.L., Chaires B., Yatsunyk L.A. Biophysical and X-ray structural studies of the (GGGTT)3GGG G-quadruplex in complex with *N*-methyl mesoporphyrin IX. *PLoS ONE*. **2020**, *15(11): e0241513*.

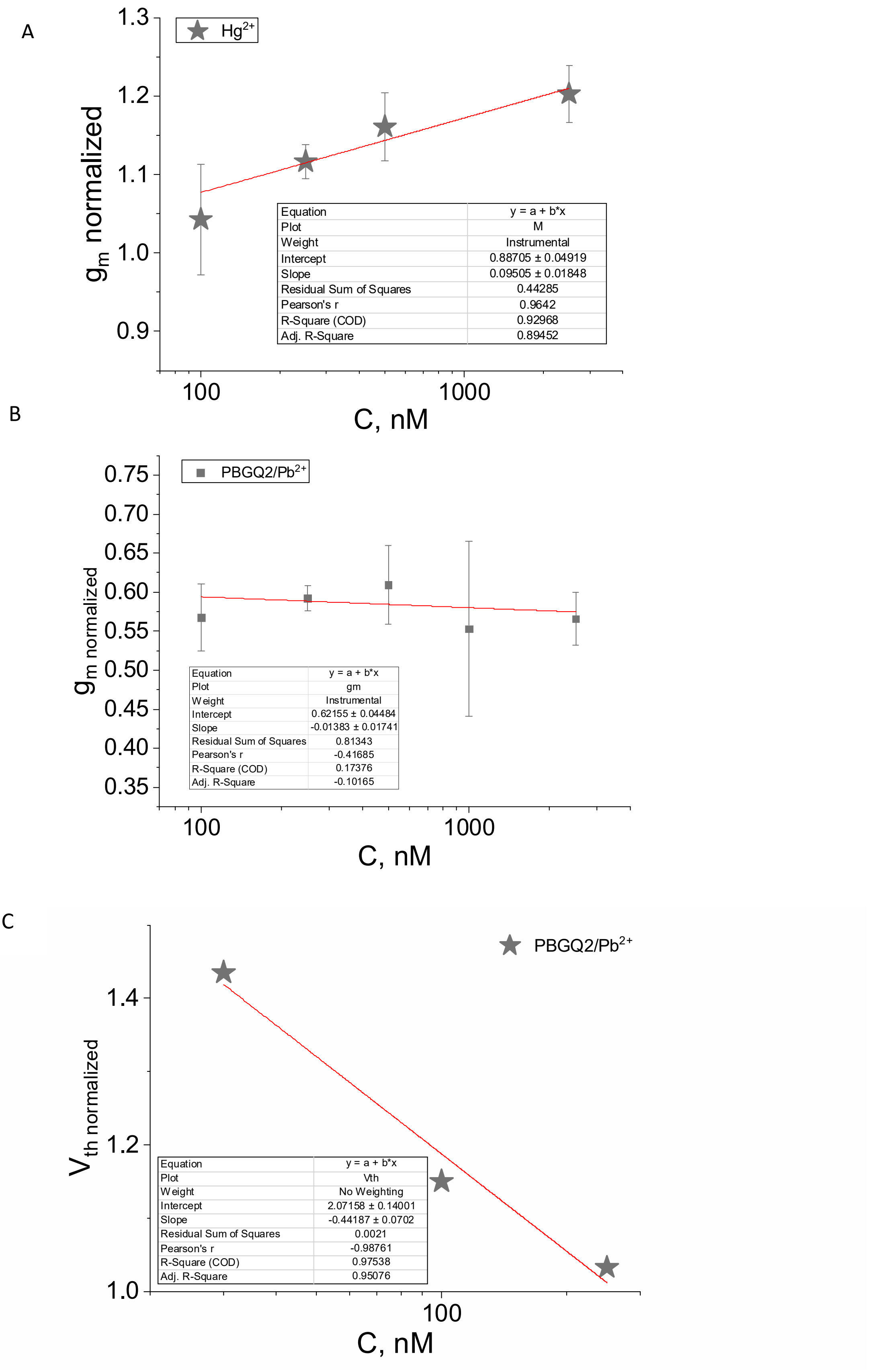

Figure S15. Dependence of the EGOFET sensor response on the target Hg^{2+} (A) and Pb^{2+} (B, C) cations concentration for $g_{m\ normalized}$ (A, B) and $V_{th\ normalized}$ (C) measured for the EGOFET modified with the aptamer HgTT specific to Hg^{2+} (A) the aptamer PbGQ2 specific to Pb^{2+} (B, C) with a confidence interval of 0.95

Table S1. Regression equations parameters, including intercepts and calibration slopes of the calibration curves of the EGOFET devices with corresponding aptamers for Hg2+ and Pb2+

Calibration curve for device, Aptamer / Cation, electrical parameter	Regression equations parameters	
	Intercept	Slope
HgTT / Hg^{2+}, g_m	0.88705 ± 0.04919	0.09505 ± 0.01848
PBGQ2 / Pb^{2+}, g_m	0.62155 ± 0.04484	-0.01383 ± 0.01741
PBGQ2 / Pb^{2+}, V_{th}	2.07158 ± 0.14001	-0.44187 ± 0.0702

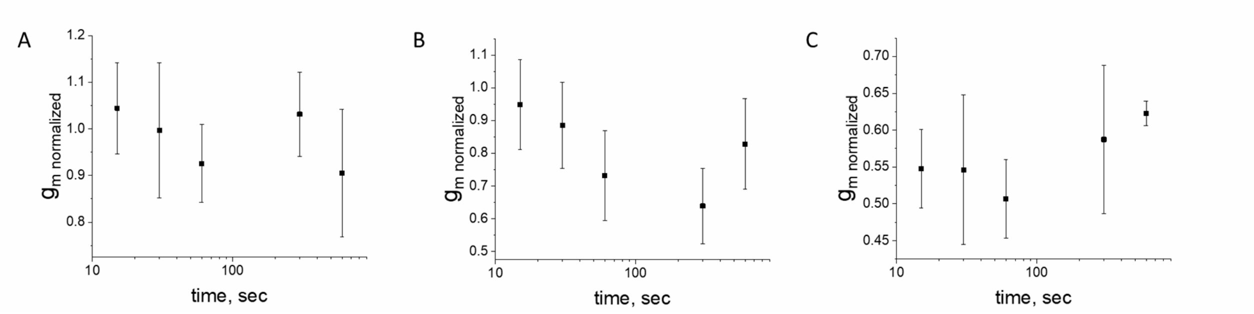

Figure S16. Time-dependent response of the EGOFET sensor based on the PbGQ2 aptamer to (A) Pb^{2+} (C=30 nM), (B) Cd^{2+} (C=30 nM) solutions in tap water, (C) to bare tap water
